# Sexual Dysfunctions in Breastfeeding Females: Systematic Review and Meta-Analysis

**DOI:** 10.3390/jcm14030691

**Published:** 2025-01-22

**Authors:** Darya Smetanina, Shouq Alnuaimi, Afra Alkaabi, Meera Alketbi, Elshimaa Hamam, Hanin Alkindi, Mahra Almheiri, Rouda Albasti, Hajar Almansoori, Mahra Alshehhi, Shamsa Al Awar, Yauhen Statsenko, Kornelia Zaręba

**Affiliations:** 1 Department of Radiology, College of Medicine & Health Sciences (CMHS), United Arab Emirates University, Al Ain P.O. Box 15551, United Arab Emirates; daryasm@uaeu.ac.ae (D.S.); e.a.statsenko@uaeu.ac.ae (Y.S.); 2 College of Medicine & Health Sciences (CMHS), United Arab Emirates University, Al Ain P.O. Box 15551, United Arab Emirates; shouqalnuaimi2002@gmail.com (S.A.); 201811752@uaeu.ac.ae (A.A.); 201814125@uaeu.ac.ae (M.A.); alshimaabdulghany202@gmail.com (E.H.); 201807797@uaeu.ac.ae (H.A.); 201913099@uaeu.ac.ae (M.A.); r.albasti9@gmail.com (R.A.); almansoorihajar@hotmail.com (H.A.); mahra.khaled@outlook.com (M.A.); 3 Department of Obstetrics & Gynecology, College of Medicine & Health Sciences (CMHS), United Arab Emirates University, Al Ain P.O. Box 15551, United Arab Emirates; sawar@uaeu.ac.ae

**Keywords:** sexual dysfunctions, sexuality, sexual health, breastfeeding, lactation, postpartum, meta-analysis, women’s health

## Abstract

**Background:** The prevalence of sexual dysfunctions varies from 35.5% to over 80% among postpartum women. Controversy exists regarding the risk factors for female sexual dysfunction (FSD) in the postpartum period. It remains unclear whether breastfeeding types contribute to the development of FSDs differently. **Aims:** The primary goal of this meta-analysis was to explore the role of baby feeding practices in developing sexual dysfunctions in women. **Methods:** We conducted a systematic literature search using the biomedical databases Scopus, CINAHL, Embase, the Web of Science, and PubMed/Medline. We looked for peer-reviewed, original studies written in English, Polish, and Arabic and published between January 2000 and June 2023. We included publications that reported scores in sexuality domains assessed with the Female Sexual Dysfunction Index (FSFI) and any sexuality-related issues during postpartum. The FSFI scores were combined in a meta-analysis using the random-effects inverse-variance model. Other findings were synthesized with a narrative review. **Results:** Eighteen articles met the eligibility criteria for the systematic review and meta-analysis. Sexual dysfunctions were detected in all the women, irrespective of the feeding type. Better overall sexual functioning was reported among women using complementary feeding than among those who breastfed exclusively or used baby bottles: 22.16, 95% CI [21.68; 22.65]; 21.61, 95% CI [20.27; 22.95]; and 20.18, 95% CI: [20.93; 61.30], respectively. Slightly lower scores were reported in all the FSFI subscales in exclusively breastfeeding women compared to those using the complementary method. **Conclusions:** Breastfeeding females experience difficulties in sexual life during the postpartum period, irrespective of the feeding type. These findings can help in designing preventive measures for tackling postpartum sexual dysfunctions in women.

## 1. Introduction

According to the World Health Organization (WHO), good sexual health encompasses “physical, emotional, mental, and social well-being in relation to sexuality” [1]. The DSM-V defines sexual dysfunction as “a clinically significant disturbance in a person’s ability to respond sexually or to experience sexual pleasure” [2]. A disturbance in a female’s sexual life is regarded as a female sexual dysfunction (FSD) [3].

The prevalence of sexual dysfunctions differs across studies, ranging from 20% to almost 50% [4,5,6,7,8]. Among postpartum women, the rate varies from 35.5% to over 80% [9,10]. Considerable heterogeneity in reporting the prevalence could be linked to methodological differences across studies. The projects use different questionnaires, cover various populations, and assess sexual health at different time points following delivery. Moreover, psychological and socio-cultural aspects could hinder research. Controlling for confounders is another issue in reporting the precise prevalence of FSDs. For example, the mode of delivery and breastfeeding seem to be the most important biological factors influencing sexual health after giving birth [11,12]. However, the data regarding the occurrence of sexual dysfunction among breastfeeding women are ambiguous and vary from 20% to 80% [10,12,13].

After childbirth, women may experience disturbances in various aspects of sexual function [14]. The divergence from pre-pregnancy sexuality can occur at both psychosexual and biological levels. The disruption can manifest in any phase of the female sexual response cycle, as described by Basson [15], and lead to decreased desire, reduced frequency of sexual intercourse, a lack of lubrication, pain, and other issues [16,17]. In the postpartum period, the most common type of dysfunction is decreased lubrication (85.6%), followed by the loss of desire (69.7%) and pain disorders (62.9%) [9].

Multiple factors account for changes in sexual behavior and physical discomfort during intercourse [14,18,19]. The causes include hormonal shifts, adjustment to a new social role, physical traumas during childbirth, and breastfeeding. A single dysfunction can be caused by an interplay between the systems of the human body and the environment [20]. For example, dyspareunia is more common in women who have had vaginal delivery, compared to those who had an elective cesarian [18]. Dyspareunia is also associated with decreased estrogen levels and increased prolactin concentrations in breastfeeding women, which lead to vaginal dryness [21,22]. Moreover, hormonal changes can result in the loss of desire associated with estrogen release, a drop in androgens, fatigue, and sleep deprivation [15,23,24]. The hypothalamic–pituitary–adrenal and hypothalamic–pituitary–gonadal axes work differently in the postpartum period compared to the pre-pregnancy period, and fluctuations in corresponding hormones can amplify the effect of individual hormonal shifts [20].

It remains unclear whether breastfeeding contributes to the development of FSDs. Dyspareunia, problems with lubrication, and a low libido have been extensively studied [9]. However, while some publications highlighted a higher prevalence of pain during intercourse in nursing mothers compared to non-breastfeeding females [25,26], others did not report any notable difference in the occurrence rate of this dysfunction. Similarly, controversy exists over changes in desire and sexual satisfaction after giving birth and vaginal atrophy [12,27]. Lev-Sagie et al. reported a high prevalence of postpartum atrophy (up to 62%) but did not confirm the correlation between dyspareunia and vaginal atrophy [28].

The prevention of FSDs is challenging due to the lack of clear guidelines on defining a threshold for disorders [29]. The absence of ideal diagnostic tools hinders the timely counseling of women about dealing with fluctuations in the frequency and quality of sexual activity [30]. Still, many symptoms remain subjective, and women may treat deviations as the norm. As a result, FSD cases may be under-reported. These factors complicate the identification of women at risk of disturbances in sexual health postpartum. The introduction of the Female Sexual Function Index (FSFI) helped to unify the assessment of sexual health and research in this field. The survey measures sexual function in six domains: desire, arousal, lubrication, orgasm, satisfaction, and pain [31]. However, the tool provides diagnostic criteria only for overall sexual dysfunction and desire hypoactive disorders.

Recent systematic reviews (SRs) have examined postpartum FSDs. Still, the findings contradict each other due to methodological differences and ethical concerns at a particular study site. The studies focused on the types of delivery, complications during childbirth, and the psychosocial determinants of FSDs. The research on sexual health in breastfeeding women lacked an explicit stratification of the study participants by the baby feeding type [26,32,33]. The authors classified the mothers as either breastfeeding or non-breastfeeding. However, a complete list of criteria defining infant-feeding practices includes breastfeeding, exclusive, predominant feeding, and bottle-feeding [34]. Another limitation of the available research was the small sample size: the number of participants rarely exceeded 500 women. SRs and meta-analyses (MA) may help to overcome the limitations of the original studies. The proposed SR and MA will be the first study examining the relationship between breastfeeding types and FSDs.

## 2. Materials and Methods

### 2.1. Objectives

The primary goal of this MA was to explore the role of baby feeding practices in developing sexual dysfunctions in women. The secondary objectives were as follows:

1. Calculate the pooled mean scores of the FSFI total and its subscales, according to the different types of infant feeding.
2. Summarize other issues related to sexuality in breastfeeding women.

### 2.2. Methodology

This review and MA focused on sexual health in generally healthy breastfeeding women. This publication was prepared following the 2020 Preferred Reporting Items for Systematic Reviews and Meta-Analyses (PRISMA) checklist, which is available online as Supplementary Table S1 in the Supplementary File. The study protocol was registered in PROSPERO (CRD 42023411053). The methodology of the SR and MA was also published elsewhere [35].

#### 2.2.1. Study Design and Data Source

This study focused on the relationship between infant feeding styles and FSD, as well as other possible disturbances in sexual health that may appear during the lactation period. We conducted the MA using scores in the total FSFI and its domains among mothers practicing various types of baby feeding. We also sought information on other sexuality-related issues and systematically reviewed the data.

A systematic literature search was conducted in the biomedical databases Scopus, CINAHL, Embase, the Web of Science, and PubMed/Medline by D.S. and the principal investigator (K.Z.). We looked for peer-reviewed original studies written in English, Polish, and Arabic that were published between January 2000 and June 2023. The inception date corresponds to the first mention of the FSFI tool. Key terms were searched in the “title/abstract”, “MeSH terms”, or “keywords” fields. The last literature search was performed on 27 May 2023. The detailed search strategy is available online in Supplementary Table S2.

#### 2.2.2. Eligibility Criteria

This study analyzed females of reproductive age, starting from 15 years old, who reported at least one sexuality-related issue within two years of childbirth. For the MA, we focused on studies reporting the mean and standard deviations (mean ± SD) of the total scores of the FSFI or its domains. The publications were deemed eligible if they reported a type of breastfeeding, the time since delivery, and the age of the participants.

We included publications that reported findings about participants who were free from mental, psychological, and neurological disorders, organic pathologies of the central nervous system and reproductive organs, cerebrovascular and endocrine diseases, and any conditions that could affect sexual health (e.g., diabetes mellitus, systemic mastocytosis). We did not consider studies assessing women who had pregnancies with severe fetal abnormalities and sexual dysfunctions known before conception. We excluded dissertations, protocol papers, reviews, case studies, editorial letters, conference posters, and presentations. We analyzed randomized-control trials, cohort and cross-sectional studies.

#### 2.2.3. Selection Process

**Figure 1:**
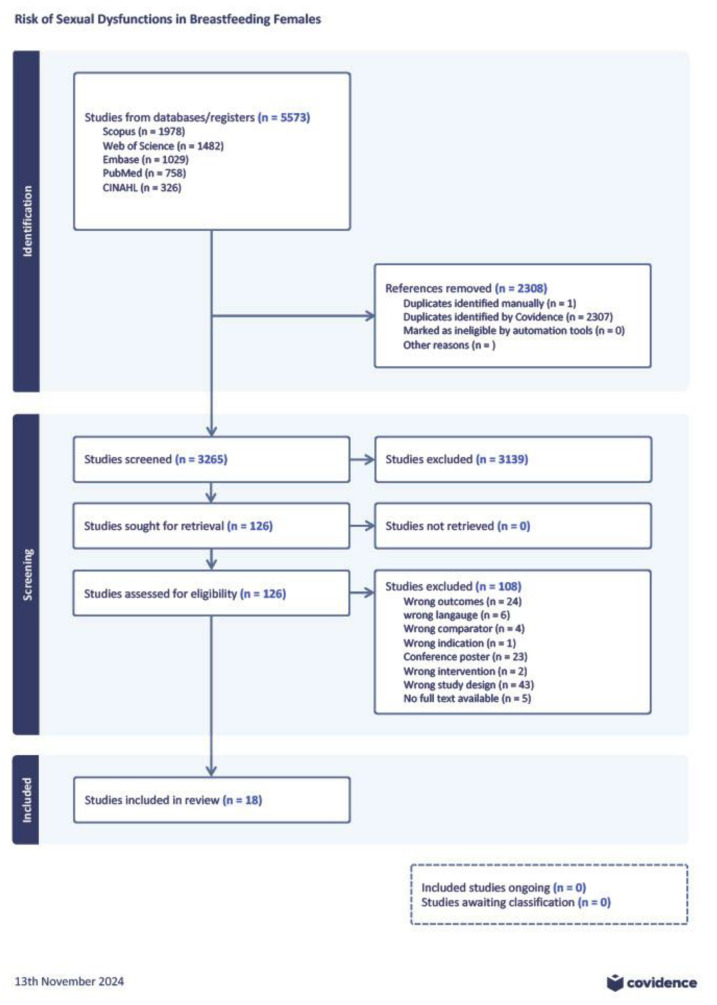
PRISMA flowchart and decision-making process for including studies in the analysis.

Records matching the search strings were uploaded to the systematic review software Covidence (Veritas Health Innovation, 2021, https://www.covidence.org, accessed on 12 June 2023) for automatic deduplication and blinded screening. Four reviewers (S.A., A.A., M.A., and E.H.) screened titles and abstracts independently by voting “yes”, “no”, or “maybe” in the software. In case of any disagreements, D.S. and K.Z. discussed whether the paper met the eligibility criteria. The same process was applied to evaluate the full texts of the publications. The results of the literature screening were automatically generated and depicted with a PRISMA flowchart (Figure 1).

#### 2.2.4. Data Collection Process

The team members extracted data into a pre-defined Microsoft Excel spreadsheet. D.S. created the spreadsheet, which K.Z. approved. Four reviewers (H.A., M.A., R.A., and H.A.) performed the data extraction independently. D.S. verified the data entered in the template.

The table consisted of two tabs. The first section included information about the FSFI scores in breastfeeding females. In this part, the data items included general information about the article: the first author’s name, year of publication, publication title, authors’ conflicts of interest, and the country where the study was conducted. The extracted data also included details about the methodology and study cohort: the study design, aims/objectives, the inclusion and exclusion criteria, the number of participants in each feeding group, age, and the time since delivery, expressed as the mean ± SD. The final set of variables consisted of the time since delivery as the mean ± SD, the breastfeeding type, the FSFI domain, the score (mean), and the score (SD).

The second part of the data extraction table covered other issues relevant to sexual health during the lactation period. This section included the same data items for the general characteristics of the publications, methodology, and study cohort as the first part of the table. The variables corresponding to sexual health consisted of the time since delivery, condition type (e.g., the resumption of intercourse, vulvovaginal atrophy), the number of participants with an issue, breastfeeding characteristics, and the measures of association.

While collecting the data, we observed variability in reporting the time since delivery and the feeding method. We converted the time since delivery into the days since giving birth. The following infant feeding styles were reported in the publications: exclusive, formula, breastfeeding plus formula, breastfeeding plus complementary feeding, breastfeeding with no further specification, non-exclusive without further specifications, predominant, complementary, mixed, and artificial. The primary investigator, an obstetrics and gynecology doctor, grouped the identified feeding practices into three methods: exclusive, formula, and breastfeeding with complementary feeding. Within each feeding method, the study cohorts were numbered sequentially (e.g., cohort1, cohort2). The classification of methods is similar to the one proposed by the WHO [34].

#### 2.2.5. Risk of Bias Assessment

For the quality assessment of individual studies, we used checklists developed by the Joanna Briggs Institute [36]. We selected tools for the appraisal of analytical cross-sectional studies, cohort studies, and randomized controlled trials. Four reviewers (H.A., M.A., R.A., and H.A.) independently answered the questions from the assessment tools. The principal investigator (K.Z.) and another reviewer (D.S.) checked the answers. Any disagreements were resolved by discussing them with the reviewer who initially appraised the article. The details of the quality assessment are available in Supplementary Table S3.

#### 2.2.6. Data Synthesis

As a part of working on the first specific objective, we resorted to the “metafor” and “dmetar” packages in R software (version 2024.04.2-764). We conducted the MA using the random-effects inverse-variance model. The mean scores and SDs in the FSFI total and its domains were combined in the MA to obtain aggregated data on the severity of the FSDs. Then, we assessed the between-study heterogeneity with the “m.gen” command. To detect the studies causing a high level of variability (*I*^2^ > 75%), the “find.outliers” function was called. The identified outliers were removed from the database. Subsequently, we divided the observations into groups according to the FSFI domains and the feeding practices. The “metamean” function was applied again to produce the results from a dataset without outliers.

To illustrate the study results, we constructed forest plots with the “meta: forest” function. The forest plot contained the following information: the name of the first author of the included study, the total number of participants, the mean and the SD FSFI scores in a studied domain, the mean raw score, the confidence interval, and the weight of the study. The box next to each study refers to its weight. The rhombus corresponds to the pooled effect and the 95% confidence interval (CI) [37].

We also examined the impact of the breastfeeding type and the country where the study was conducted on the FSFI score. For this, we performed a meta-regression using the “metareg” function in R.

Working on the second objective, we analyzed the findings from the eligible studies using a narrative approach.

#### 2.2.7. Bias Assessment

We examined the publication bias among the studies included in the MA. We constructed funnel plots using the “funnel” function in the R software. The graph facilitates the visual inspection of the publication bias. Asymmetric funnel plots indicate the low precision of the effect sizes (see Supplementary Figures S8–S15). In the funnel plots, the central vertical line represents the overall effect size (the mean FSFI score), the inclined lines denote the 95% CI, and the dots represent individual studies in the MA [38].

## 3. Results

### 3.1. Literature Search

We retrieved 5573 studies from the biomedical databases. After deduplication, 3265 publications remained for the title and abstract screening. One hundred and twenty-six articles were identified for the full-text evaluation against the eligibility criteria. The final dataset included 18 publications. The findings from eight studies were incorporated into the meta-analysis. The remaining publications contributed to the qualitative analysis of postpartum sexual health.

### 3.2. The Characteristics of the Studies Included in the Meta-Analysis

The studies included in the MA were conducted in Iran [39,40,41,42], Brazil [43,44], Canada [45], and Japan [46]. The sample size varied from 27 to 355 women (See Table 1). The ages of the participants were consistent across the publications. The time since giving birth did not exceed six months. The classification of the breastfeeding practices was not uniform across the studies. In total, we identified nine types of infant feeding. Six publications reported scores in all the domains of the FSFI questionnaire. Saotome et al. considered the overall sexual dysfunction in breastfeeding women [46]. The quality of the studies ranged from low to high (Supplementary Table S3).

### 3.3. Scores in FSFI Domains Irrespective of Infant-Feeding Practices

We analyzed the pooled scores for the total FSFI and its subscales, irrespective of the feeding practices (Supplementary Figures S1–S6). The results in the desire domain were below the clinical cut-off point for diagnosing a desire disorder: 3.08, 95% CI [2.92; 3.24], compared to 5 points on the scale.The other subscales do not have a threshold for healthy sexual functioning. However, the highest mean score was reported for the satisfaction domain: 4.12, 95% CI [3.79; 4.46]. In this subscale, the scores can vary from 0.8 to 6. The total FSFI score can range from 2 to 36. In our sample, the overall score was 21.97, 95% CI [21.09; 22.84]. In most clinical research, the cut-off point for diagnosing sexual dysfunctions was 26.55 out of 36.

### 3.4. Sexual Function in Women Choosing Distinct Infant-Feeding Methods

#### 3.4.1. Desire

**Figure 2:**
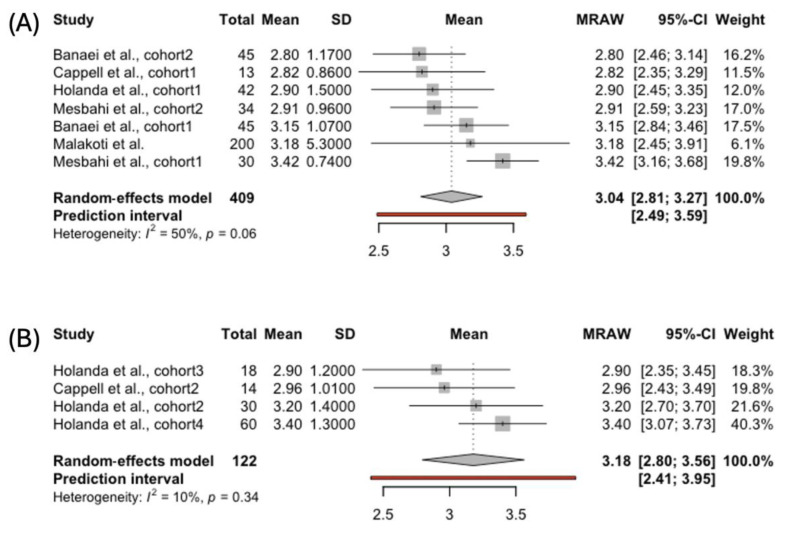
Forest plot showing pooled score in the FSFI Desire domain in women choosing (**A**) exclusive [40,41,42,43,45] and (**B**) complemented breastfeeding [43,45].

The aggregated FSFI desire score was also calculated for 409 women breastfeeding exclusively and 122 females using complementary feeding (Figure 2). The first group exhibited slightly lower scores on the desire subscale than the second group: 3.04, 95% CI [2.81; 3.27], compared to 3.18, 95% CI [2.80; 3.56]. Desire is the only FSFI domain with a clinically validated cut-off point of five for confirming a hypoactive desire disorder. Both the groups of women met the criteria for diagnosing dysfunction in the desire domain. No significant heterogeneity was observed among the studies in either group. The funnel plots were also symmetric, indicating the absence of a publication bias.

#### 3.4.2. Arousal

**Figure 3:**
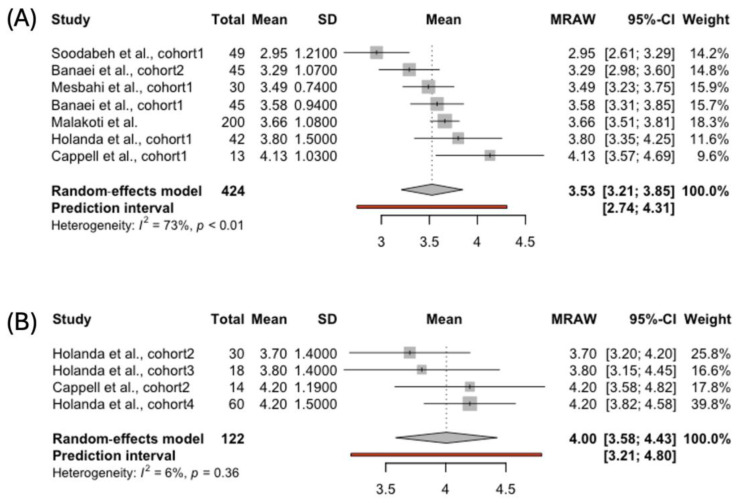
Forest plot presenting pooled score in the FSFI Arousal domain in women choosing (**A**) exclusive [39,40,41,42,43,45] and (**B**) complemented breastfeeding [43,45].

We did not receive enough studies reporting the FSFI arousal scores among women using bottle-feeding. The analysis included 424 participants breastfeeding exclusively and 122 women using complementary breastfeeding (Figure 3). In the latter group, the pooled FSFI arousal score was higher than that of women feeding their babies solely with breast milk: 4.00, 95% CI [3.58; 4.43], vs. 3.53, 95% CI [3.21; 3.85], respectively. The heterogeneity between the studies was greater for the publications reporting arousal scores among exclusively breastfeeding mothers (*I*² = 73%, *p* < 0.01). We did not observe any publication bias in either group.

#### 3.4.3. Orgasm

**Figure 4:**
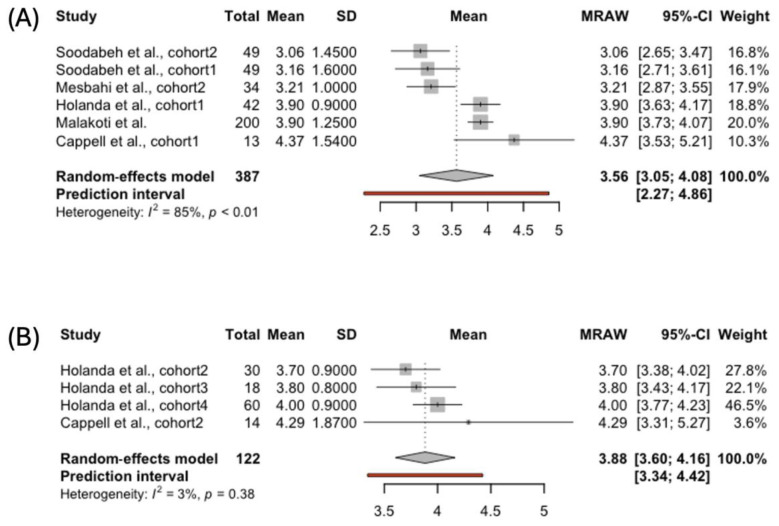
Forest plot presenting pooled score in the FSFI Orgasm domain in women choosing (**A**) exclusive [39,41,42,43,45] and (**B**) complemented breastfeeding [43,45].

The FSFI scores for the orgasm subscale were reported for 387 women breastfeeding exclusively and 122 females using complementary feeding (Figure 4). The pooled results for the orgasm domain indicated lower scores in the women feeding their babies solely with human milk compared to those complementing breastfeeding: 3.56, 95% CI [3.05; 4.08], vs. 3.88, 95% CI [3.60; 4.16], respectively. The heterogeneity was high for the studies reporting orgasm scores among women breastfeeding exclusively (*I*^2^ = 85%, *p* < 0.01). Therefore, the results should be interpreted with caution.

#### 3.4.4. Lubrication

**Figure 5:**
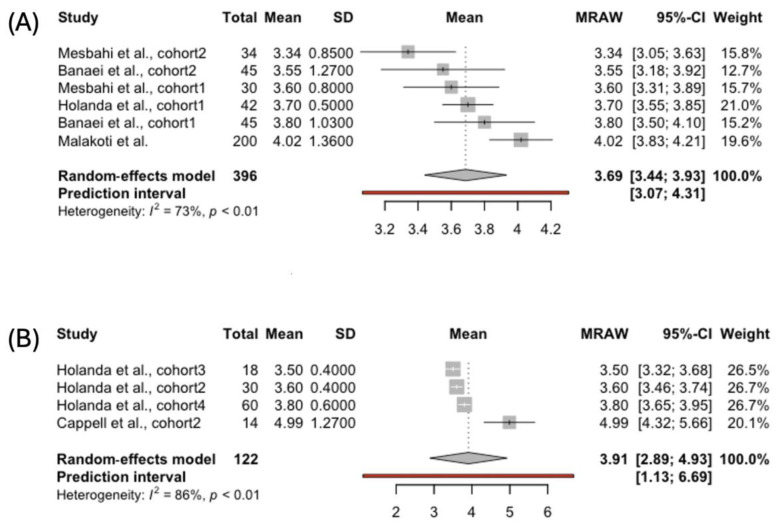
Forest plot presenting pooled score in the FSFI Lubrication domain in women choosing (**A**) exclusive [40,41,42,43] and (**B**) complemented breastfeeding [43,45].

The FSFI lubrication domain was assessed in 396 women breastfeeding exclusively and in 122 females who were using complementary feeding (Figure 5). The pooled score was lower among the mothers exclusively breastfeeding than among those using mixed feeding: 3.69, 95% CI [3.44; 3.93], compared to 3.91, 95% CI [2.89; 4.93], respectively. A high between-study heterogeneity was observed in both the groups. However, no publication biases were evident on the funnel plots.

#### 3.4.5. Pain

**Figure 6:**
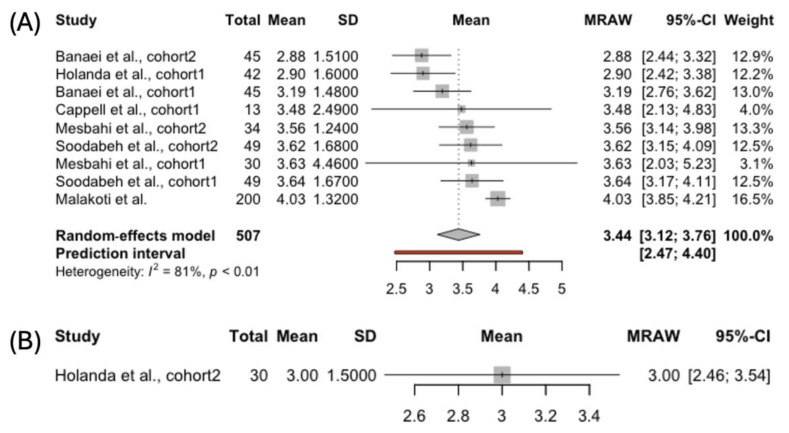
Forest plot presenting pooled score in the FSFI Pain domain in women choosing (**A**) exclusive [39,40,41,42,43,45] and (**B**) complemented breastfeeding [43].

The removal of outliers led to excluding the studies that reported scores on the FSFI pain domain in women opting for complementary feeding (Figure 6). Among 507 women who were breastfeeding exclusively, the random-effects model yielded a pooled score of 3.44, 95% CI [3.12; 3.76]. The studies analyzed were highly heterogeneous (*I*² = 81%, *p* < 0.01). The funnel plot exhibited slight asymmetry, suggesting the potential presence of a bias.

#### 3.4.6. Satisfaction

**Figure 7:**
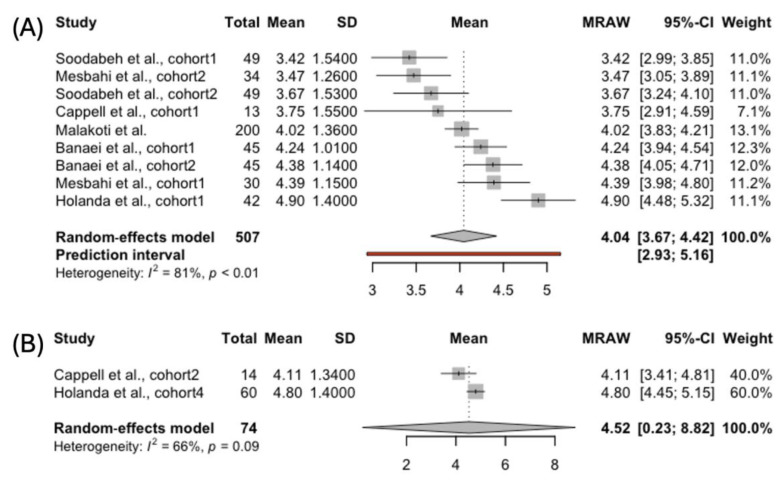
Forest plot presenting pooled score in the FSFI Satisfaction domain in women choosing (**A**) exclusive [39,40,41,42,43,45] and (**B**) complemented breastfeeding [43,45].

Satisfaction was measured among 507 exclusively breastfeeding women and 74 women using complementary feeding (Figure 7). This domain received the highest score in both the groups compared to the other FSFI subscales. The exclusively breastfeeding women scored lower than those using complementary feeding: 4.04 95% CI [3.67; 4.42] vs. 4.52 95% CI [0.23; 8.82]. The heterogeneity was high among the studies reporting data for the exclusively breastfeeding group (*I*² = 81%, *p* < 0.01), while in the second group, it was moderate. However, only two studies were included in the analysis, complicating the generalizability of these findings beyond the analyzed studies.

#### 3.4.7. Overall Sexual Function

**Figure 8:**
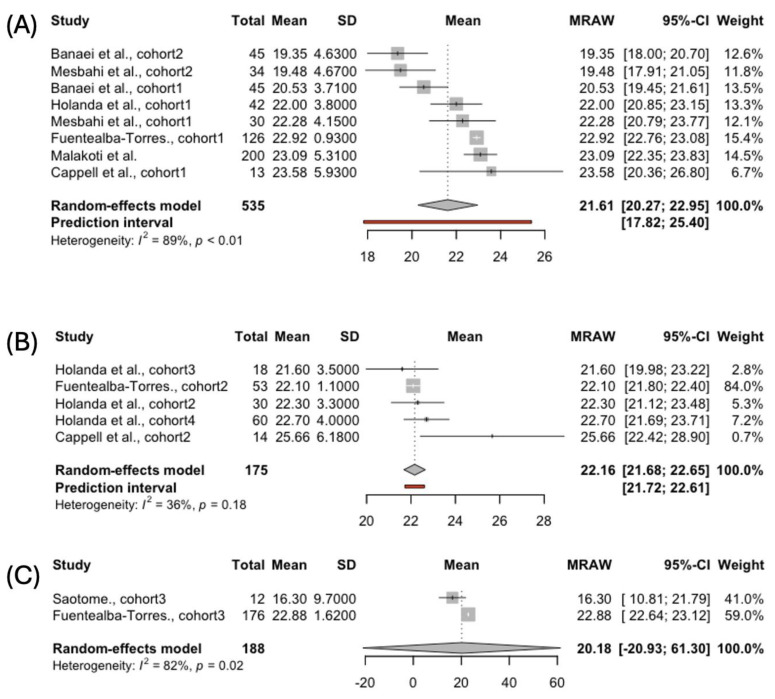
Forest plot presenting pooled score in the total FSFI score in women choosing (**A**) exclusive [40,41,42,43,44,45], (**B**) complemented breastfeeding [43,44,45], and (**C**) formula [44,46].

Sexual dysfunction was detected in all women, regardless of the feeding type. Better sexual functioning was reported among the women using complementary feeding compared to those who breastfed exclusively or used baby bottles: 22.16, 95% CI [21.68; 22.65]; 21.61, 95% CI [20.27; 22.95]; and 20.18, 95% CI [-20.93; 61.30], respectively. Overall sexual functioning was reported in the studies examining FSDs in three groups of women: exclusively breastfeeding (n = 535), using complementary feeding (n = 175), and bottle-feeding (n = 188) (Figure 8). Complementary feeding appears to be the best option in regard to the risks for overall sexual satisfaction. A significant inter-study heterogeneity was found among the studies reporting the FSFI scores for exclusively breastfeeding women (*I*^2^ = 89%, *p* < 0.01) and those who bottle-fed (*I*^2^ = 82%, *p* = 0.02). The funnel plots did not indicate a publication bias in any of the three groups.

### 3.5. Impact of Breastfeeding and Country of the Study on the Results

Significant heterogeneity was observed in several subgroups of the MA. We explored the potential predictive effect of the breastfeeding type and the country of the study on the overall results of the MA. The analysis indicated that the type of feeding practice was not a significant predictor of the FSFI scores. The country of the study location was significantly related to the heterogeneity in the study results (see Table 2).

### 3.6. The Description of the Studies Included in a Systematic Review

We identified ten publications addressing sexuality-related issues in the postpartum period [28,32,47,48,49,50,51,52,53]. The studies were conducted in Uganda, Iran (two papers), Israel, Ireland, Sweden, Canada, Malaysia, the USA, and Spain. The sample sizes ranged from 108 to 832 participants (Table 3).

### 3.7. Resumption of Sexual Intercourse

Four studies focused on the resumption of sexual intercourse among breastfeeding women. Alum et al. reported that 82% of exclusively breastfeeding women from Uganda resumed intercourse before 6 weeks postpartum [47]. Among Spanish women, 23.10% of nursing mothers engaged in sexual activity within the same period [32]. In another study, 28.3% of breastfeeding Iranian women returned to sexual activity within a month of giving birth [27]. The publication did not provide information on whether the type of infant feeding was associated with the resumption of intercourse. In a sample of Swedish women, 75.3% of breastfeeding participants returned to sexual activity within 3 months of childbirth. In the same study, over 90% of nursing mothers had sexual relations within 6 months following delivery [49]. According to Signorello et al., breastfeeding females resumed sexual activity 0.8 weeks earlier than their non-breastfeeding counterparts [53].

Changes in sexuality were explored in four studies [15,27,50,52]. These changes included reduced desire, satisfaction, a lack of vaginal lubrication, difficulty in reaching orgasm, and overall decreased sexual functioning. The studies utilized ad hoc questionnaires to assess postpartum sexual functioning. Rezai et al. and Salamon et al. examined sexual health using the FSFI; however, the studies did not report scores in any of the domains [50,52]. As a result, these publications were excluded from the MA. According to Rezai et al., exclusive breastfeeding was identified as a risk factor for sexual dysfunction: aOR: 2.47; 95% CI [1.21; 5.03] [50]. Salamon et al. did not compare sexual issues across different feeding styles but confirmed a higher risk of FSD in breastfeeding women compared to those who were not breastfeeding: aOR: 2.24; 95% CI [1.03; 4.85] [52]. Heidari et al. found no significant difference between breastfeeding and bottle-feeding women regarding sexual desire and satisfaction before and after pregnancy [27]. Both groups did not exhibit notable differences in experiencing orgasm before conception and after delivery. O’Malley et al. compared vaginal lubrication and a loss of interest in sexual activity between breastfeeding and non-breastfeeding women [48]. At six months postpartum, breastfeeding was linked to changes in these two domains.

Two authors investigated the relationship between breastfeeding and pain during intercourse [51,53]. Rosen et al. modeled changes in dyspareunia levels up to 24 months postpartum. The study did not confirm the effect of breastfeeding on the dyspareunia trajectory postpartum [54]. Signorello et al. identified breastfeeding as a strong predictor of pain at the first instance of postpartum intercourse, at 3 and 6 months after delivery: OR: 2.2; 95% CI [1.4; 3.2]; OR: 2.7; 95% CI [1.8; 4.1]; and OR: 4.4; 95% CI [2.7; 7.0] [53]. A single study examined the incidence of vulvovaginal atrophy in breastfeeding vs. non-breastfeeding women. The disorder was more prevalent in breastfeeding women than in those who were not breastfeeding [28].

## 4. Discussion

### 4.1. Impact of Infant-Feeding Practices on Female Sexual Health

Breastfeeding is crucial for the healthy development of newborns, both biologically and psychologically. From the maternal perspective, Stone and Smith define breastfeeding as a “sexual and reproductive health right” [54]. Regrettably, the postpartum period may be associated with the occurrence of sexual disorders [55]. Breastfeeding may further intensify these disorders due to both biological factors arising from hormonal changes and fatigue resulting from frequent night awakenings [19]. However, the study presented did not yield clear results in this respect.

The first part of the current MA identified sexual dysfunction in the overall sample of postpartum women, irrespective of the feeding type. Women breastfeeding exclusively had lower scores in all the FSFI domains (desire, arousal, orgasm, lubrication, and satisfaction) than those choosing complementary feeding. The findings align with recent research by Sun et al., which reported a high incidence of sexual dysfunctions in both groups [55]. According to Fuentealba-Torres et al., sexual dysfunction was present in 58.3% of breastfeeding females and was linked to a low quality of life [12]. In the presented sample, the total FSFI score was 21.97, 95% CI [21.09; 22.84]. Clinicians use a score of 26.55 out of 36 to differentiate between women with and without sexual dysfunction. The results imply that the participants experienced difficulties in their sexual lives during the postpartum period, irrespective of the feeding type. This may be due to hormonal changes that still occur even when the breasts are stimulated infrequently [56]. It remains unclear whether the concentration of hormones is the same in mothers breastfeeding exclusively and those using complementary feeding. For formula-feeding mothers, FSDs may arise due to the fatigue women experience from needing to wake up frequently to prepare the infant’s food [57]. Mariman et al. did not find any difference in the level of fatigue between mothers who breastfeed exclusively and those who bottle-feed [58].

In the second part of the MA, the mothers who exclusively breastfed had slightly lower scores on the desire subscale than those using complementary feeding: 3.04, 95% CI [2.81; 3.27], vs. 3.18, 95% CI [2.80; 3.56]. Both groups met the criteria for diagnosing dysfunction in the desire domain. The results align with research conducted by Kayner et al. on 121 nursing women with lactation amenorrhea who predominantly reported low desire [58]. Desire is primarily linked to the psychological aspect of sexuality [59]. According to Basson’s theory, we can hypothesize that women are less inclined to engage in sexual intercourse when experiencing the vaginal atrophy associated with exclusive lactation [28,60].

In the presented study, the FSFI arousal score was higher in the females who were complementing breastfeeding compared to the women feeding solely with human milk: 4.95%, 95% CI [3.58; 4.43], vs 3.53, 95% CI [3.21; 3.85], respectively. Arousal is the biological effect of desire that depends on genital and hormonal well-being [59]. Changes occurring during lactation may influence these systems. Moreover, sexual arousal is also related to sexual desire, which can impact the final biological effect. The same biological aspect may play a role in the ability to achieve orgasm among exclusively breastfeeding women. The pooled results for the orgasm domain were lower in the women feeding their babies solely with human milk compared to the females who were complementing breastfeeding: 3.56, 95% CI [3.05; 4.08], vs. 3.88, 95% CI [3.60; 4.16], respectively. The results presented align with Fuentealba-Torres et al., who reported a high prevalence of sexual dysfunctions in the arousal (83%) and orgasm domains (76.3%) among breastfeeding females [12].

The pooled score for the lubrication domain was lower among the exclusively breastfeeding mothers compared to those using mixed feeding: 3.69, 95% CI [3.44; 3.93], vs. 3.91, 95% CI [2.89; 4.93], respectively. Vaginal lubrication is primarily linked with estrogen levels, which are lower in breastfeeding women, particularly in those practicing exclusive lactation [61]. Elevated prolactin levels, resulting from breast stimulation, inhibit estrogen secretion, leading to vulvovaginal atrophy, dryness, and subsequent dyspareunia.

The MA revealed lower scores on the satisfaction subscale in the exclusively breastfeeding women compared to the mothers using complementary feeding: 4.04, 95% CI [3.67; 4.42], vs 4.52, 95% CI [0.23; 8.82]. On the other hand, Fuentealba-Torres et al. reported that among all the FSFI domains, sexual satisfaction was low in only 50.9% of participants, being less predisposed to decrease than the other domains, which reached 70–80% of cases [12]. Supposedly, this domain also addresses Basson’s theory, in which sexual intercourse is merely a part of all the aspects of sexual satisfaction, involving relational dimensions such as mutuality, interpersonal closeness, romance, cohabitation, and the expression of feelings [62,63].

### 4.2. Cultural Context of Postpartum Sexual Health

The MA indicated a significant impact of the country of study on the FSFI scores. The results reflect cultural differences in reporting issues related to sexuality. Specific dysfunctions are described as “culture-bound syndromes” and manifest in various forms and with different degrees across cultures and countries [64]. Women may feel shy and embarrassed discussing their sexual health with their physicians, particularly in conservative countries [8]. Moreover, the existing tools for assessing sexual health were developed in the West and, despite being validated, may be less sensitive and specific to other cultural contexts [65]. Consequently, the true prevalence of FSDs remains unknown, and studies are conducted on small sample sizes.

In traditional societies, sexuality is a taboo topic and is studied only in the social and medical contexts. Socio-cultural norms, traditions, and superstitions have shaped women’s attitudes towards feeding practices and the resumption of intercourse after childbirth. For instance, Christian and Druze mothers showed higher rates of exclusive breastfeeding compared to Muslim mothers living in Israel [66]. In some countries, an early return to sexual activity is encouraged, as it is believed to offer a protective effect against extramarital affairs and sexually transmitted diseases. However, resuming sexual contact within six weeks after childbirth is associated with unwanted pregnancies, vaginal dryness, and puerperal infection [67]. Thus, social norms may impact female well-being both positively and negatively.

Sexual health is influenced by religious codes of conduct and customs such as maintaining virginity and female genital cutting in some countries [68]. A recent systematic review revealed that low sexual desire was linked to female genital mutilation among Egyptian women. The same review emphasized that poor partner performance and an aversion to looking at or touching genitalia were risk factors for FSDs among women in Iran. Socio-cultural factors were determinants of sexual well-being in Turkey, Morocco, and Palestine [69]. In these countries, postpartum sexual health should be examined alongside the impact of traditional practices on sexuality.

The findings suggest that new approaches should be adopted for the assessment of sexual health in women. Physicians should ask more questions about the potential occurrence of sexual dysfunctions. Some, such as vaginal atrophy, can be managed with simple vaginal treatment [70].

### 4.3. The Implication of the Study Results for Clinical Practice

The study results should be carefully interpreted due to significant heterogeneity among the studies reporting information about dysfunction in specific domains. A high *I*^2^ index was noted among the publications detailing function in the domains of desire, arousal, orgasm, and pain in women who were exclusively breastfeeding. Considerable heterogeneity was reported for the studies reporting lubrication and satisfaction scores among women engaging in exclusive and supplemented breastfeeding. Similarly, notable heterogeneity was noted across the publications regarding the total FSFI scores among women breastfeeding exclusively and those using formula.

The significant variability among the studies may arise for several reasons. First, the studies lacked consistent definitions for the types of breastfeeding, with ten feeding practices identified across eight publications. A standardized approach to reporting infant-feeding types could mitigate ambiguity in presenting the findings. Second, some subgroups had either extremely low or extremely high scores in the FSFI domains. Although these cases were not flagged as outliers, they could have influenced the results of the MA. Third, studies with small sample sizes tend to exhibit greater heterogeneity. Despite high heterogeneity in several subgroups, the present study provides valuable insights into the risk factors for postpartum FSDs.

### 4.4. Policy Recommendation

Our findings highlighted the importance of raising awareness among women, their partners, and medical practitioners on possible sexual dysfunctions among breastfeeding women. This goal can be achieved through the early education of expectant mothers and their partners, initiated during pregnancy in birthing schools, in hospitals after delivery, and following discharge within the community. Furthermore, patients should be screened by nurses or doctors during postpartum visits.

Public health initiatives should focus on promoting maternal programs, including OTC treatments for vaginal dryness and social support for fatigued mothers. Additionally, raising awareness among mothers, their partners, and close people about the possibility of sexual dysfunction will enhance the quality of life and plans for procreation.

Moreover, healthcare professionals should undergo training in the diagnosis and treatment of sexual dysfunctions. The FSFI seems to be the most useful diagnostic tool for screening in this group of patients.

#### 4.4.1. Limitations

The meta-analysis possesses limitations that should be considered and addressed in future similar works:

- The search strategy did not yield a sufficient number of publications reporting sexual dysfunctions among women who resorted to bottle-feeding. Therefore, we did not calculate pooled scores in individual sexuality domains for this group of women. This limitation suggests a direction for future original studies. The impact of bottle-feeding should also be examined extensively for two reasons. First, women expressing milk for bottle-feeding may still experience dissatisfaction with their appearance and breast discomfort, which can lead to an unsatisfactory sexual experience. Second, women may have a low desire for intercourse due to the fatigue associated with frequent awakenings to prepare formula for the baby.
- The initial analysis revealed a high heterogeneity index. To address significant between-study variability, we identified and removed outliers. Omitting studies with extreme values may reduce the generalizability of the study findings beyond the results of this work. Still, the current review is the first step in discovering the effect of feeding practices on female sexual health in postpartum. To confirm the link between FSD and breastfeeding, original studies should analyze more participants.
- Since the number of included studies was low, we could not adjust the findings for time since delivery. However, this information could provide valuable insights into the dynamics of sexual functioning in the postpartum period.
- It remains challenging to compare results of the current MA with findings from other papers due to a limited number of publications focusing on sexual health in breastfeeding females.

To overcome these limitations, the current MA should be updated regularly. The Cochrane Collaboration recommends updating the search strategy every two years. Refining the literature search will identify new relevant studies. Consequently, adding new sources will provide clinicians with stronger evidence about the impact of feeding practices on FSDs.

#### 4.4.2. Strengths

- The work was prepared in accordance with the PRISMA guidelines and registered in the PROSPERO database.
- The research produced aggregate scores across all the areas of sexual functioning using the FSFI scale. Earlier SR and MA focused on overall sexual health data. Nevertheless, understanding changes within each domain is crucial for creating effective counseling approaches for couples expecting a baby.
- The FSFI scores were calculated separately for each type of feeding practice. The findings reflect a possible relationship between hormonal changes in lactating women and their sexual function postpartum.
- No significant publication biases were detected across the studies. The funnel plots were symmetric in most of the subgroups, indicating that the studies had similar effect sizes.
- The SR covered other possible changes in sexual health in breastfeeding females. The findings revealed a range of issues women face in the postpartum period that require attention from healthcare specialists.

## 5. Conclusions

Breastfeeding females experience difficulties in their sexual lives during the postpartum period, regardless of the type of feeding. The prevalence of sexual dysfunctions (considering both the overall score and all domains) is slightly higher among patients who are exclusively breastfeeding. Early diagnosis and the prevention of postpartum sexual dysfunctions should be viewed as crucial steps in maintaining sexual health for breastfeeding women.

## Figures and Tables

**Table 1:** Characteristics of studies included in meta-analysis.

Author, Year	Country	Total Sample Size	Patient’s Age	Time Since Delivery	Breastfeeding Type	Studied FSFI Domains
Soodabeh et al., 2020 [39]	Iran	98	29.65 ± 5.66	4.12 ± 1.61 months	Exclusive.	Total; Libido (desire); Arousal; Lubrication; Orgasm; Satisfaction; Pain.
Banaei et al., 2018 [40]	Iran	87	24.93 ± 3.10 in the intervention group; 23.44 ± 2.64 in the control group	3.56 ± 1.58 months in the intervention group; 3.56 ± 1.80 months in the control group	Exclusive.	Total; Desire; Arousal; Lubrication; Orgasm; Satisfaction; Pain.
Cappell et al., 2020 [45]	Canada	27	31.45 ± 4.35	310.26 ± 204.26 days	Exclusive; Not exclusive.	Total; Desire; Arousal; Lubrication; Orgasm; Satisfaction; Pain.
Fuentealba-Torres et al., 2019 [44]	Brazil	355	26.5 ± 6.68	N/A	Exclusive; Predominant; Complementary.	Total
Holanda et al., 2021 [43]	Brazil	150	24.8 ± 6.4	4.3 ± 1.2 months	Exclusive; Predominant; Complemented; Mixed.	Total; Desire; Arousal; Lubrication; Orgasm; Satisfaction; Pain.
Malakoti et al. 2013 [41]	Iran	200	27.5 ± 5.2	3–6 months	Exclusive.	Total; Desire; Arousal; Lubrication; Orgasm; Satisfaction; Pain.
Mesbahi et al., 2022 [42]	Iran	64	31.2 ± 5.1 in the intervention group; 27.8 ± 5.9 in the control group	4.18 ± 1.88 months in the intervention group; 3.87 ± 1.72 in the control group	Exclusive.	Total; Desire; Arousal; Lubrication; Orgasm; Satisfaction; Pain.
Saotome et al., 2018 [46]	Japan	84	32.8 ± 4.4	N/A	Exclusive; Mixed; Formula.	Total

**Table 2:** Results of meta-regression analysis.

	Estimate	Standard Error	T-Statistics	Degree of Freedom	*p*-Value	95% CI
Intercept	4.05	0.16	24.75	75	<0.01	[3.73; 4.38]
Canada	0.21	0.32	0.64	75	0.53	[−0.44; 0.85]
Egypt	1.46	0.38	3.83	75	0.0003	[0.70; 2.22]
Iran	−0.43	0.35	−1.24	75	0.22	[−1.13; 0.26]
Japan	11.11	3.67	3.03	75	0.003	[3.81; 18.41]
Exclusive breastfeeding	−0.11	0.33	−0.33	75	0.75	[−0.77; 0.56]
Formula feeding	−0.36	0.43	−0.83	75	0.41	[−1.20;0.49]

**Table 3:** Characteristics of studies included in the systematic review.

Author, Year	Country	Sample Size	Age of Participants	Time Since Delivery	Target Condition	Feeding Practice	Key Findings
Alum et al., 2015 [47]	Uganda	374	Between 15 and 45	N/A.	Resumption of sexual intercourse after 6 weeks.	Any type of breastfeeding vs. artificial feeding; exclusive vs. non-exclusive breastfeeding.	Of the participants, 21.6% resumed intercourse within 6 weeks after giving birth. The early resumption of intercourse was associated with socio-economic factors.
Heidari et al., 2009 [27]	Iran	456	Between 20 and 35	2–6 months.	Resumption of sexual intercourse after 6 weeks; reduced desire; reduced satisfaction; not experiencing orgasm.	Breastfeeding vs. bottle-feeding.	Breastfeeding and bottle-feeding women did not have a significant difference in sexual health postpartum.
Lev-Sagie et al., 2020 [28]	Israel	329	Between 23 and 40	3–16 weeks.	Vulvovaginal atrophy.	Breastfeeding vs. non-breastfeeding (not specified).	Vulvovaginal atrophy was associated with breastfeeding status.
O’Malley et al., 2018 [48]	Ireland	832	18 and above	6 and 12 months.	Lack of vaginal lubrication; loss of interest in sexual activity.	Breastfeeding vs. non-breastfeeding (not specified).	Breastfeeding and pre-existing dyspareunia were risk factors for issues in sexual health at 6 months postpartum.
Radestad et al., 2008 [49]	Sweden	2342	15 and above	12 months.	Intercourse at over 3 and over 6 months after giving birth.	Breastfeeding at 2 months and 6 months vs. not breastfeeding (not specified).	Breastfeeding women had 1.6 OR of resuming intercourse at over 3 months postpartum.
Rezaei et al., 2017 [50]	Iran	380	18 and above	3–5 months.	Total FSFI score.	Exclusive breastfeeding.	Exclusive breastfeeding was significantly associated with sexual dysfunction (adjusted OR: 2.47; 95% CI [1.21–5.03]).
Rosen et al., 2022 [51]	Canada	582	29 ± 4.4	Up to 2 years.	Change from moderate to minimal dyspareunia.	Breastfeeding at 3 months (not specified).	Breastfeeding did not predict a dyspareunia class.
Salamon et al., 2020 [52]	Malaysia	249	28.99 ± 6.07	4–6 months.	Overall sexual dysfunction.	Breastfeeding (not specified).	Breastfeeding was a risk factor for sexual dysfunction (adjusted OR: 2.24; 95% CI [1.03–4.85]).
Signorello et al., 2001 [53]	USA	615	N/A	8.1 ± 3.5 weeks; 3 months; 6 months.	Pain at the first instance of postpartum sexual intercourse; pain on sexual intercourse at 3 and 6 months postpartum.	Breastfeeding vs. non-breastfeeding (not specified).	Breastfeeding women were 4 times as likely to experience dyspareunia compared to non-breastfeeding mothers.
Triviño-Juárez et al., 2018 [32]	Spain	552	32.18 ± 5.36	6 weeks.	Resumption of sexual intercourse at 6 weeks; decline in sexual intercourse.	Breastfeeding (not specified).	Breastfeeding was a determinant of dyspareunia. However, nursing was not linked to the resumption of intercourse or a decline in sexual activity.

## Data Availability

No new data were created for this study.

## Supplementary Materials

The following supporting information can be downloaded at: https://www.mdpi.com/article/10.3390/jcm14030691/s1, Table S1. Search strategy; Table S2. PRISMA 2020 checklist; Table S3. Quality assessment of individual studies; Figure S1. Arousal in Postpartum Women; Figure S2. Desire in postpartum women; Figure S3. Orgasm in postpartum women; Figure S4. Lubrication in postpartum women; Figure S5. Pain in postpartum women; Figure S6. Satisfaction in Postpartum Women; Figure S7. Overall sexual function in postpartum women; Figure S8. Funnel plots exploring publications bias in studies reporting arousal (A), desire (B), orgasm (C), lubrication (D), pain (E), satisfaction (F), and overall sexual function (G); Figure S9. Funnel plots depicting publication bias for studies on arousal in women choosing (A) exclusive and (B) complemented breastfeeding; Figure S10. Funnel plots depicting publication bias for studies on desire in women choosing (A) exclusive and (B) complemented breastfeeding; Figure S11. Funnel plots depicting publication bias for studies on orgasm in women choosing (A) exclusive and (B) complemented breastfeeding; Figure S12. Funnel plots depicting publication bias for studies on lubrication in women choosing (A) exclusive and (B) complemented breastfeeding; Figure S13. Funnel plots depicting publication bias for studies on pain in women choosing (A) exclusive and (B) complemented breastfeeding; Figure S14. Funnel plots depicting publication bias for studies on satisfaction in women choosing (A) exclusive and (B) complemented breastfeeding; Figure S15. Funnel plots depicting publication bias for studies on overall FSFI score in women choosing (A) exclusive, (B) complemented breastfeeding, and (C) bottle-feeding.

## Abbreviations

The following abbreviations are used in the manuscript:

CI	confidence interval
FSD	female sexual dysfunction
FSFI	Female Sexual Function Index
MA	meta-analysis
SD	standard deviation
SR	systematic review
WHO	World Health Organization
