# Supplementary material for: Sexual Dysfunctions in Breastfeeding Females: Systematic Review and Meta-Analysis
Source: J Clin Med. 2025 Jan 22;14(3):691. doi: 10.3390/jcm14030691 (PMC11818559; doi:10.3390/jcm14030691)
Supplement: Supplementary file 1 [file jcm-14-00691-s001.zip › jcm-3386706-supplementary.pdf]

SUPPLEMENTARY MATERIAL

Sexual Dysfunctions in Breastfeeding Females: Systematic Review and Meta-Analysis

Darya Smetanina [1], Shouq Alnuaimi [2], Afra Alkaabi [2], Meera Alketbi [2], Elshimaa Hamam [2], Hanin Alkindi [2], Mahra Almheiri [2], Rouda Albasti [2], Hajar Almansoori [2], Mahra Alshehhi [2], Shamsa Al Awar [3], Yauhen Statsenko [1] and Kornelia Zaręba [3,*]

[1] Department of Radiology, College of Medicine & Health Sciences (CMHS), United Arab Emirates University, P.O. Box 15551, Al Ain, United Arab Emirates; daryasm@uaeu.ac.ae (D.S.); e.a.statsenko@uaeu.ac.ae (Y.S.)

[2] College of Medicine & Health Sciences (CMHS), United Arab Emirates University, P.O. Box 15551 Al Ain, United Arab Emirates; shouqalnuaimi2002@gmail.com (S.A.); 201811752@uaeu.ac.ae (A.A.); 201814125@uaeu.ac.ae (M.A.); alshimaabdulghany202@gmail.com (E.H.); 201807797@uaeu.ac.ae (H.A.); 201913099@uaeu.ac.ae (M.A.); r.albasti9@gmail.com (R.A.); almansoorihajar@hotmail.com (H.A.); mahra.khaled@outlook.com (M.A.)

[3] Department of Obstetrics & Gynecology, College of Medicine & Health Sciences (CMHS), United Arab Emirates University, P.O. Box 15551, Al Ain, United Arab Emirates; sawar@uaeu.ac.ae

* Correspondence: kornelia3@poczta.onet.pl; Tel.: +48-662-051-602

Supplementary Table S1. PRISMA 2020 checklist

Section and Topic	Item #	Checklist item	Location where item is reported
**TITLE**			
Title	1	Identify the report as a systematic review.	Page 1; lines 1-3
**ABSTRACT**			
Abstract	2	See the PRISMA 2020 for Abstracts checklist.	
**INTRODUCTION**			
Rationale	3	Describe the rationale for the review in the context of existing knowledge.	Pages 1-3; lines 43 - 112
Objectives	4	Provide an explicit statement of the objective(s) or question(s) the review addresses.	Page 3, lines 113 – 119
**METHODS**			
Eligibility criteria	5	Specify the inclusion and exclusion criteria for the review and how studies were grouped for the syntheses.	Page 3, lines 149 - 157
Information sources	6	Specify all databases, registers, websites, organisations, reference lists and other sources searched or consulted to identify studies. Specify the date when each source was last searched or consulted.	Page 3, lines 134 – 135
Search strategy	7	Present the full search strategies for all databases, registers and websites, including any filters and limits used.	Suppl. Table S1
Selection process	8	Specify the methods used to decide whether a study met the inclusion criteria of the review, including how many reviewers screened each record and each report retrieved, whether they worked independently, and if applicable, details of automation tools used in the process.	Page 4, lines 158 - 166
Data collection process	9	Specify the methods used to collect data from reports, including how many reviewers collected data from each report, whether they worked independently, any processes for obtaining or confirming data from study investigators, and if applicable, details of automation tools used in the process.	Pages 5-6, lines 170 - 199
Data items	10a	List and define all outcomes for which data were sought. Specify whether all results that were compatible with each outcome domain in each study were sought (e.g. for all measures, time points, analyses), and if not, the methods used to decide which results to collect.	Page 5-6, lines 175 - 190
	10b	List and define all other variables for which data were sought (e.g. participant and intervention characteristics, funding sources). Describe any assumptions made about any missing or unclear information.	N/A
Study risk of bias assessment	11	Specify the methods used to assess risk of bias in the included studies, including details of the tool(s) used, how many reviewers assessed each study and whether they worked independently, and if applicable, details of automation tools used in the process.	Page 6, lines 209 - 230
Effect measures	12	Specify for each outcome the effect measure(s) (e.g. risk ratio, mean difference) used in the synthesis or presentation of results.	Page 6, lines 212 - 213
Synthesis methods	13a	Describe the processes used to decide which studies were eligible for each synthesis (e.g. tabulating the study intervention characteristics and comparing against the planned groups for each synthesis (item #5)).	Page 6, lines 2012-213
	13b	Describe any methods required to prepare the data for presentation or synthesis, such as handling of missing	N/A

Section and Topic	Item #	Checklist item	Location where item is reported
		summary statistics, or data conversions.	
	13c	Describe any methods used to tabulate or visually display results of individual studies and syntheses.	Page 6, lines 220-225
	13d	Describe any methods used to synthesize results and provide a rationale for the choice(s). If meta-analysis was performed, describe the model(s), method(s) to identify the presence and extent of statistical heterogeneity, and software package(s) used.	Page 6, lines 210 - 219
	13e	Describe any methods used to explore possible causes of heterogeneity among study results (e.g. subgroup analysis, meta-regression).	Page 6, lines 226-228
	13f	Describe any sensitivity analyses conducted to assess robustness of the synthesized results.	Page 6, lines 215-218
Reporting bias assessment	14	Describe any methods used to assess risk of bias due to missing results in a synthesis (arising from reporting biases).	Page 7, lines 232 - 237
Certainty assessment	15	Describe any methods used to assess certainty (or confidence) in the body of evidence for an outcome.	N/A
**RESULTS**			
Study selection	16a	Describe the results of the search and selection process, from the number of records identified in the search to the number of studies included in the review, ideally using a flow diagram.	Page 7, lines 240 - 245
	16b	Cite studies that might appear to meet the inclusion criteria, but which were excluded, and explain why they were excluded.	N/A
Study characteristics	17	Cite each included study and present its characteristics.	Pages 15 – 16
Risk of bias in studies	18	Present assessments of risk of bias for each included study.	Suppl. Table 3
Results of individual studies	19	For all outcomes, present, for each study: (a) summary statistics for each group (where appropriate) and (b) an effect estimate and its precision (e.g. confidence/credible interval), ideally using structured tables or plots.	Figures 2 – 7
Results of syntheses	20a	For each synthesis, briefly summarise the characteristics and risk of bias among contributing studies.	N/A
	20b	Present results of all statistical syntheses conducted. If meta-analysis was done, present for each the summary estimate and its precision (e.g. confidence/credible interval) and measures of statistical heterogeneity. If comparing groups, describe the direction of the effect.	Pages 7 – 13, lines 256 – 347
	20c	Present results of all investigations of possible causes of heterogeneity among study results.	Page 13, lines 352 - 357
	20d	Present results of all sensitivity analyses conducted to assess the robustness of the synthesized results.	N/A
Reporting biases	21	Present assessments of risk of bias due to missing results (arising from reporting biases) for each synthesis assessed.	Suppl Figures S8 – S15
Certainty of evidence	22	Present assessments of certainty (or confidence) in the body of evidence for each outcome assessed.	N/A
**DISCUSSION**			
Discussion	23a	Provide a general interpretation of the results in the context of other evidence.	Pages 16 – 18,

Section and Topic	Item #	Checklist item	Location where item is reported
			lines 407 - 505
	23b	Discuss any limitations of the evidence included in the review.	Page 19, lines 545 - 553
	23c	Discuss any limitations of the review processes used.	Page 19, lines 555 - 564
	23d	Discuss implications of the results for practice, policy, and future research.	Pages 18-19, lines 506 - 541
**OTHER INFORMATION**			
Registration and protocol	24a	Provide registration information for the review, including register name and registration number, or state that the review was not registered.	Page 3, lines 124 - 125
	24b	Indicate where the review protocol can be accessed, or state that a protocol was not prepared.	Page 3, line 126
	24c	Describe and explain any amendments to information provided at registration or in the protocol.	N/A
Support	25	Describe sources of financial or non-financial support for the review, and the role of the funders or sponsors in the review.	Page 20, lines 623 – 624
Competing interests	26	Declare any competing interests of review authors.	Page 20, line 628
Availability of data, code and other materials	27	Report which of the following are publicly available and where they can be found: template data collection forms; data extracted from included studies; data used for all analyses; analytic code; any other materials used in the review.	N/A

Supplementary Table S2. Search strategy

Date: 27.05.2023

PubMed			
**Search no**	**String**	**Results**	**Notes**
1	(((((((("Breast Feeding"[Mesh]) OR ("Breast Feeding"[Title/Abstract])) OR (Breast-feeding[Title/Abstract])) OR ("Breast Feeding"[Text Word])) OR (Breast-feeding[Text Word])) OR (breastfeeding[Text Word])) OR (breastfeeding[Title/Abstract])) OR (lactat*[Title/Abstract])) OR (lactat*[Text Word])	771,408	
2	((((((((((((((((sexual dysfunction, physiological[MeSH Terms]) OR (sexual dysfunction*[Title/Abstract])) OR (sexual disorder*[Title/Abstract])) OR (sexual dysfunctions, psychological[MeSH Terms])) OR (desire[Title/Abstract])) OR (arousal[Title/Abstract])) OR (aversion[Title/Abstract])) OR (Vulvodynia[MeSH Terms])) OR (Vulvodynia[Title/Abstract])) OR (dyspareunia[Title/Abstract])) OR (dyspareunia[MeSH Terms])) OR (vaginismus[MeSH Terms])) OR (vaginismus[Title/Abstract])) OR (libido[Title/Abstract])) OR (libido[MeSH Terms])) OR (Lubrication[Title/Abstract])) OR (sexual abstinence[MeSH Terms])) OR (sexual abstinence[Title/Abstract])	44,468	
3	#1 AND #2	758	Filter: publications starting from year 2000
**Scopus**			
1	(TITLE-ABS-KEY (breast AND feeding) OR TITLE-ABS-KEY (breastfeeding) OR TITLE-ABS-KEY (breast-feeding) OR TITLE-ABS-KEY (lactat*))	450,309	
2	(TITLE-ABS-KEY (sexual AND dysfunction*) OR TITLE-ABS-KEY (	491,208	

	sexual AND disorder*) OR TITLE-ABS-KEY (desire) OR TITLE-ABS-KEY (arousal) OR TITLE-ABS-KEY (aversion) OR TITLE-ABS-KEY (vulvodynia) OR TITLE-ABS-KEY (dyspareunia) OR TITLE-ABS-KEY (vaginismus) OR TITLE-ABS-KEY (sexual AND pain) OR TITLE-ABS-KEY (libido) OR TITLE-ABS-KEY (lubrication) OR TITLE-ABS-KEY (sexual AND abstinence))		
3	#1 AND #2	1,966	Filter: publications starting from year 2000; language English, Polish
**Web of Science**			
1	Breast Feeding (Topic) OR Breast-feeding (Topic) OR breastfeeding (Topic) OR lactation (Topic) OR lactating (Topic)	272,351	
2	sexual dysfunction (Topic) OR sexual disorder (Topic) OR desire (Topic) OR aversion (Topic) OR arousal (Topic) OR Vulvodynia (Topic) OR dyspareunia (Topic) OR vaginismus (Topic) OR sexual pain (Topic) OR libido (Topic) OR lubrication (Topic) OR sexual abstinence (Topic)		
3	#1 AND #2	1428	Filter: publications starting from year 2000; language English, Polish
**EMBASE**			
1	'breast feeding'/exp OR 'breast feeding' OR breastfeeding:ti,ab,kw OR 'breast feeding':ti,ab,kw OR lactation:ti,ab,kw OR lactating:ti,ab,kw	140,078	
2	'sexual dysfunction'/exp OR 'sexual dysfunction' OR 'psychosexual disorder':ti,ab,kw OR 'female sexual dysfunction':ti,ab,kw OR desire:ti,ab,kw OR arousal:ti,ab,kw OR 'aversive	205,314	

	behavior':ti,ab,kw OR vulvodynia:ti,ab,kw OR dyspareunia:ti,ab,kw OR vaginism:ti,ab,kw OR 'sexual pain':ti,ab,kw OR libido:ti,ab,kw OR orgasm:ti,ab,kw OR 'sexual abstinence':ti,ab,kw		
3	#1 AND #2	1,029	Filter: publications starting from year 2000
**CINAHL**			
1	MW Breast Feeding OR AB Breast Feeding OR TI Breast Feeding OR TI breastfeeding OR AB breastfeeding OR MW lactation OR TI lactation OR AB lactation	40,207	
2	MW Sexual Dysfunction, Female OR TI sexual dysfunction OR AB sexual dysfunction OR TI sexual disorder OR AB sexual disorder OR TI desire OR AB desire OR TI aversion OR AB aversion OR TI arousal OR AB arousal	34,806	
3	TI Vulvodynia OR AB Vulvodynia OR TI dyspareunia OR AB dyspareunia OR TI vaginismus OR AB vaginismus OR TI sexual pain OR AB sexual pain OR TI libido OR AB libido OR TI lubrication OR AB lubrication	3,628	
4	TI sexual abstinence OR AB sexual abstinence	198	
5	#2 OR #3 OR #4	37,775	
6	#1 AND #5	326	Filters: Year of publication: 2000-2023<br><br>Language: English

Supplementary Table S3. Quality assessment of individual studies

Question Author	Were the criteria for inclusion in the sample clearly defined?	Were the study subjects and the setting described in detail?	Was the exposure measured in a valid and reliable way?	Were objective, standard criteria used for measurement of the condition?	Were confounding factors identified?	Were strategies to deal with confounding factors stated?	Were the outcomes measured in a valid and reliable way?	Was appropriate statistical analysis used?	Overall "yes" answers
Cappell et al., 2020 [45]	yes	yes	yes	yes	no	no	yes	yes	6
Fuentealba-Torres et al., 2019 [44]	yes	yes	yes	yes	yes	yes	yes	yes	8
Holanda et al., 2021 [43]	yes	yes	yes	yes	yes	no	yes	yes	7
Malakoti et al., 2013 [41]	no	yes	yes	yes	no	no	yes	yes	5
Saotome et al., 2018 [46]	yes	yes	yes	yes	yes	yes	yes	yes	8
Alum et al., 2015 [47]	no	no	yes	no	no	no	yes	yes	3
Heidari et al., 2009 [27]	no	yes	yes	no	no	no	yes	yes	4
Rezaei et al., 2017 [50]	yes	yes	yes	yes	yes	yes	yes	yes	8

Salamon et al., 2020 [52]	partially	yes	yes	yes	yes	yes	yes	Yes	7.5

S3.1. Cross-sectional studies

S3.2. Cohort studies

Question / Author	Were the two groups similar and recruited from the same population?	Were the exposures measured similarly to assign people to both exposed and unexposed groups?	Was the exposure measured in a valid and reliable way?	Were confounding factors identified?	Were strategies to deal with confounding factors stated?	Were the groups/participants free of the outcome at the start of the study (or at the moment of exposure)?	Were the outcomes measured in a valid and reliable way?	Was the follow up time reported and sufficient to be long enough for outcomes to occur?	Was follow up complete, and if not, were the reasons to loss to follow up described and explored?	Were strategies to address incomplete follow up utilized?	Was appropriate statistical analysis used?	Overall "yes" answers
Lev-Sagie et al., 2020 [28]	yes	yes	yes	yes	no	N/A	yes	yes	partially	no	yes	7.5
O'Malley et al., 2018 [48]	yes	yes	yes	yes	yes	No	yes	yes	yes	yes	yes	10
Radestad et al., 2008 [49]	Yes	Yes	Yes	yes	Yes	no	Yes	partially	No	No	yes	7.5
Rosen et al., 2022 [51]	Yes	Yes	Yes	Yes	Yes	No	Yes	Yes	Yes	Yes	yes	10
Signorello et al., 2001 [53]	yes	yes	yes	yes	yes	No	yes	Yes	Yes	No	yes	9
Triviño-Juárez et al., 2017 [32]	yes	yes	yes	yes	yes	no	yes	yes	no	no	yes	8

S3.3 Control-trials

Question / Author	Was true randomization used for assignment of participants to treatment groups?	Was allocation to treatment groups concealed?	Were treatment groups similar at the baseline?	Were participants blind to treatment assignment?	Were those delivering the treatment blind to treatment assignment?	Were treatment groups treated identically other than the intervention of interest?	Were outcome assessors blind to treatment assignment?	Were outcomes measured in the same way for treatment groups?	Were outcomes measured in a reliable way?	Was follow up complete and if not, were differences between groups in terms of their follow up adequately described and analysed?	Were participants analysed in the groups to which they were randomized?	Was appropriate statistical analysis used?	Was the trial design appropriate and any deviations from the standard RCT design (individual randomization, parallel groups) accounted for in the conduct and analysis of the trial?	Overall "yes" answers
Soodabeh et al., 2020 [39]	Yes	N/A	yes	N/A	no	yes	no	yes	yes	yes	yes	yes	partially	8.5
Banaei et al., 2018 [40]	yes	n/a	yes	n/a	no	yes	no	yes	yes	yes	yes	yes	partially	8.5
Mesbahi et al., 2022 [42]	yes	yes	yes	yes	n/a	n/a	yes	yes	yes	yes	yes	yes	yes	11

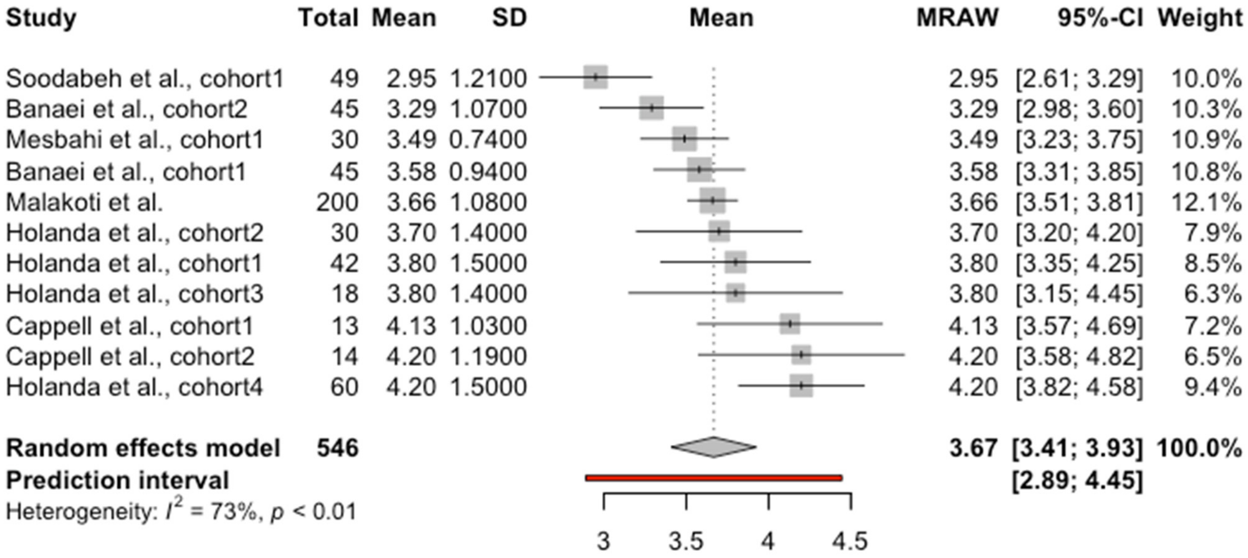

Supplementary Figure S1. Forest plot presenting pooled score in arousal domain in Postpartum Women

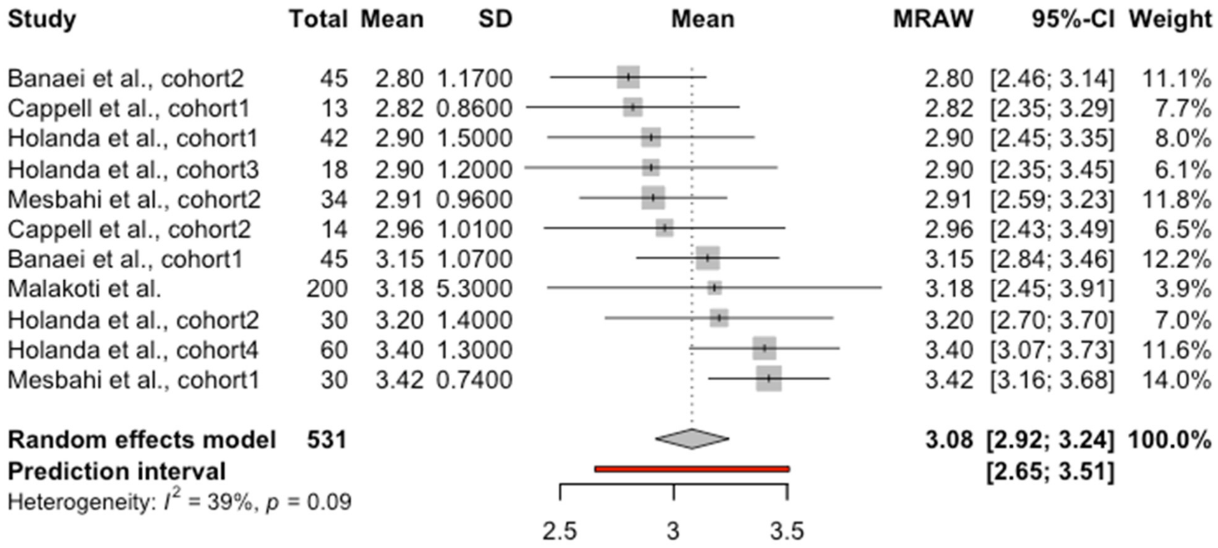

Supplementary Figure S2. Forest plot presenting pooled score in desire domain in postpartum women

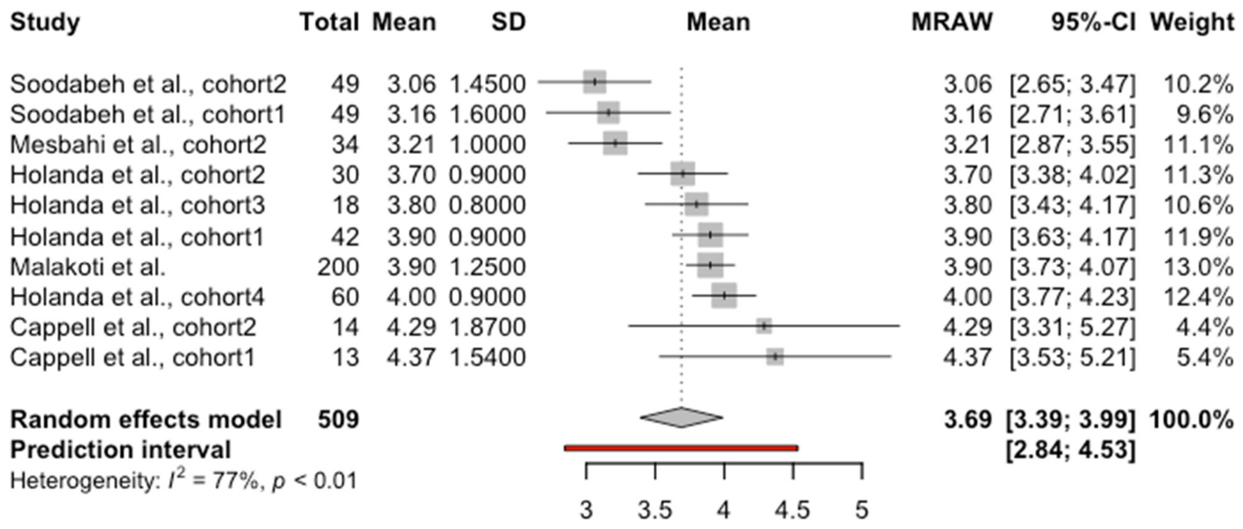

Supplementary Figure S3. Forest plot presenting pooled score in orgasm domain in postpartum women

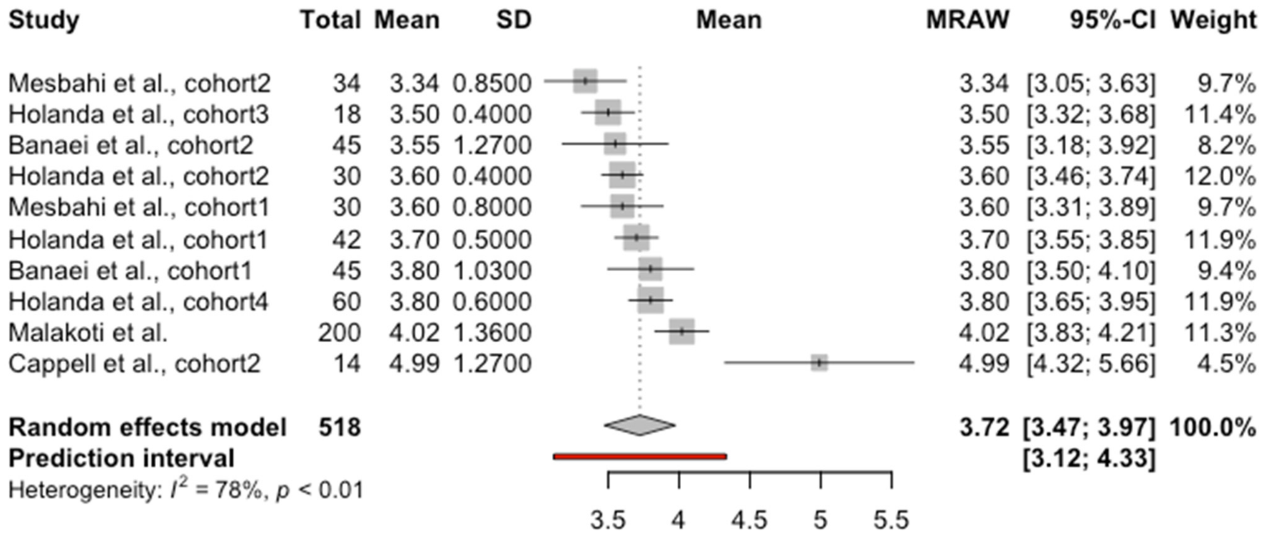

Supplementary Figure S4. Forest plot presenting pooled score in lubrication domain in postpartum women

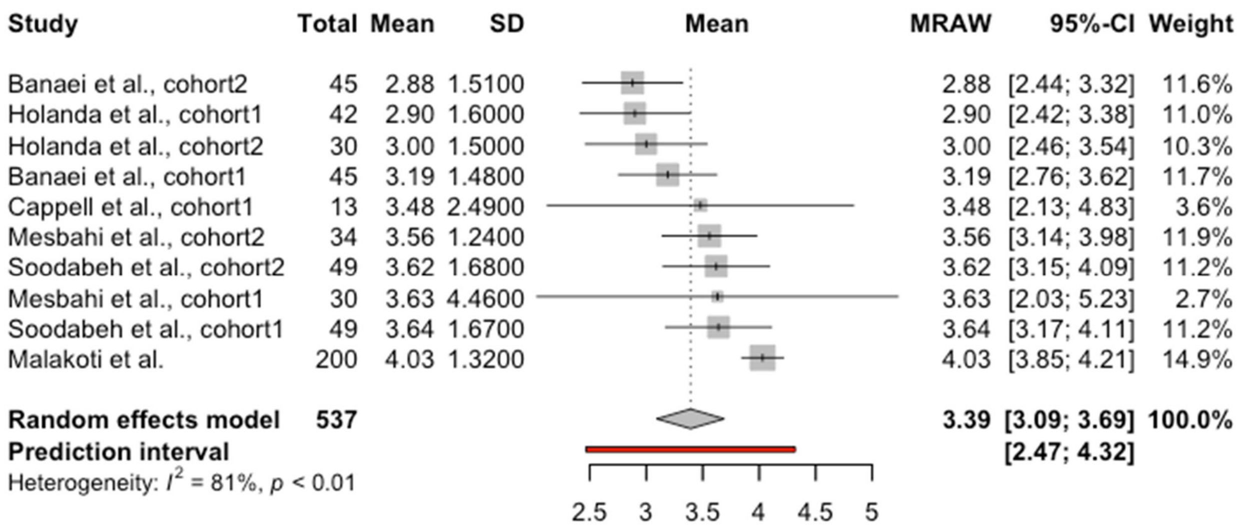

Supplementary Figure S5. Forest plot presenting pooled score in pain domain in postpartum women

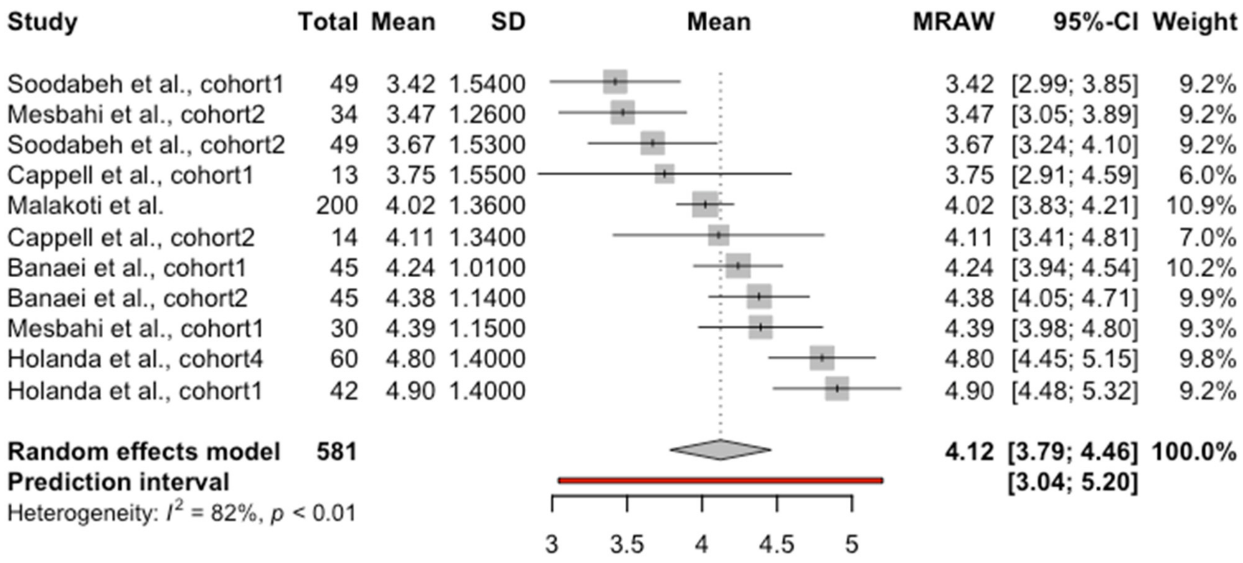

Supplementary Figure S6. Forest plot presenting pooled score in satisfaction domain in Postpartum Women

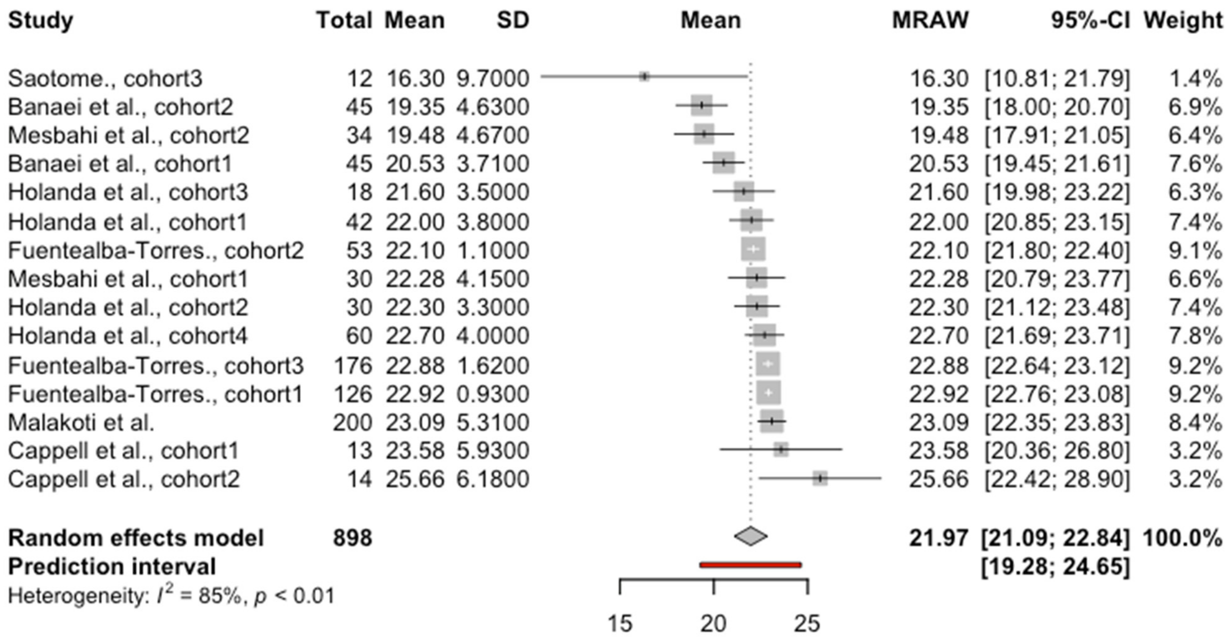

Supplementary Figure S7. Forest plot presenting pooled score of overall sexual function in postpartum women

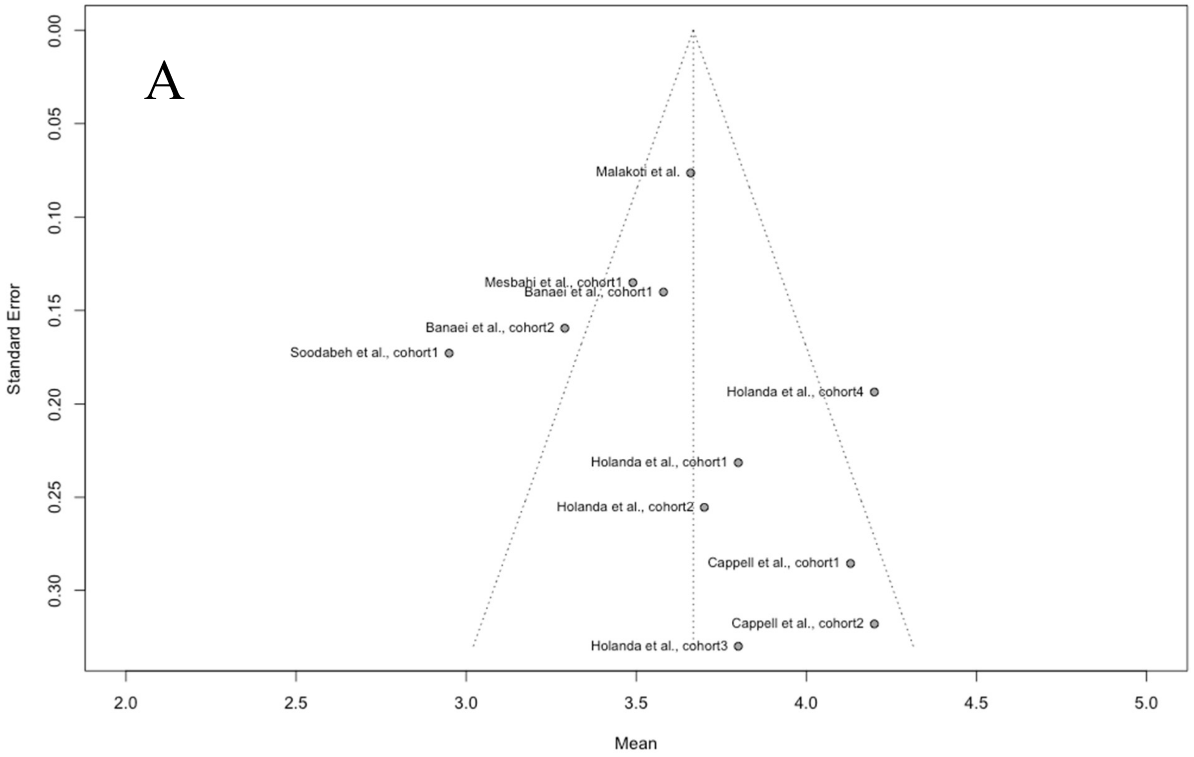

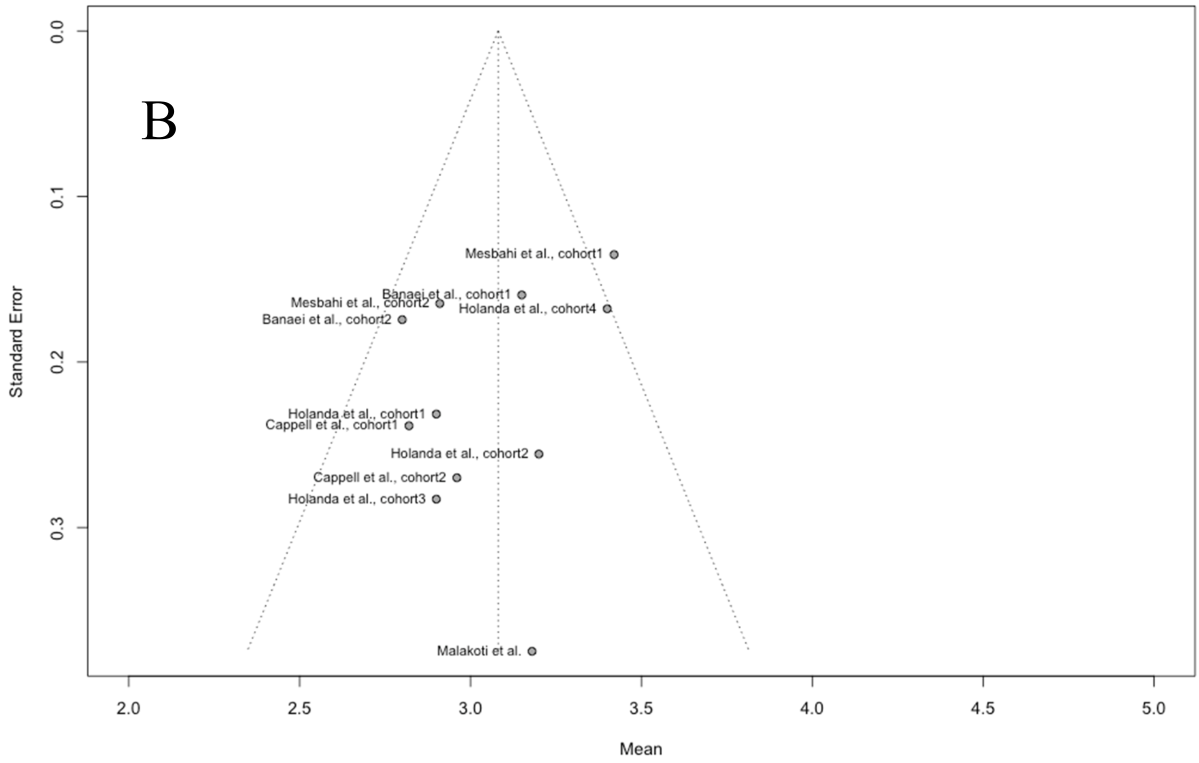
B
Standard Error
Mean
0.0
0.1
0.2
0.3
2.0
2.5
3.0
3.5
4.0
4.5
5.0
Mesbahi et al., cohort1
Banaei et al., cohort1
Mesbahi et al., cohort2
Holanda et al., cohort4
Banaei et al., cohort2
Holanda et al., cohort1
Cappell et al., cohort1
Holanda et al., cohort2
Cappell et al., cohort2
Holanda et al., cohort3
Malakoti et al.

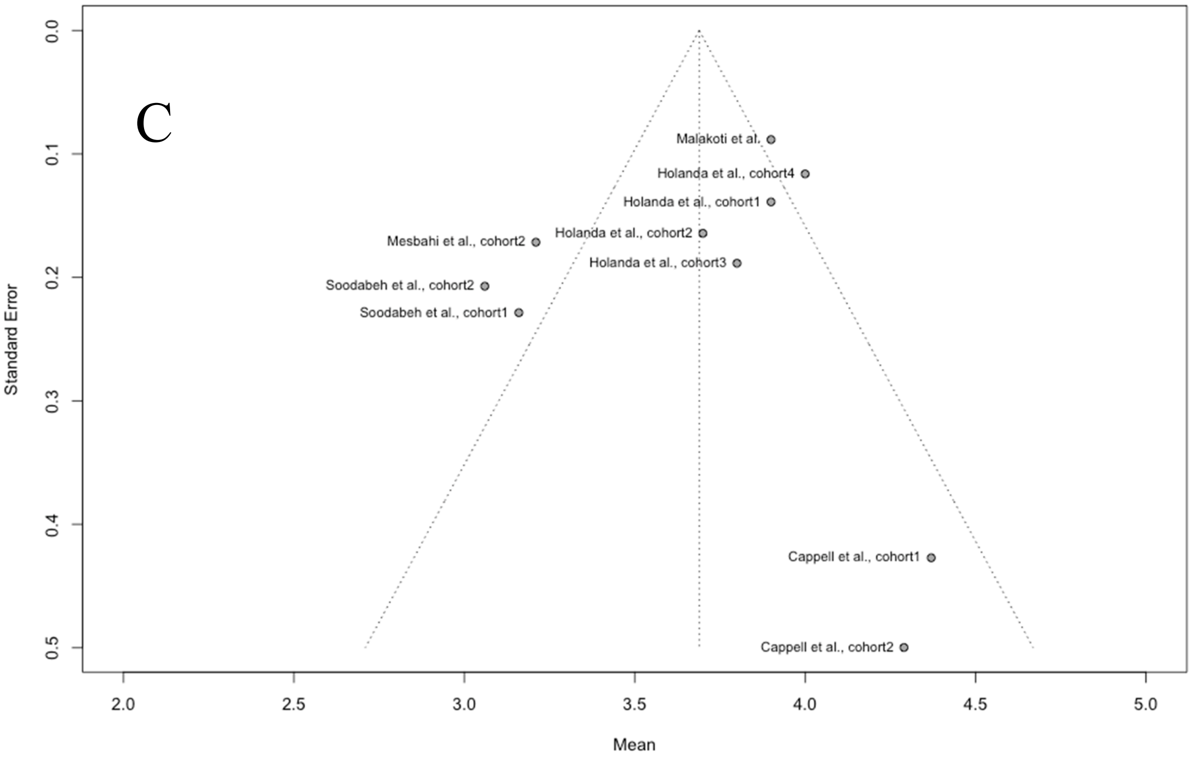
C
Malakoti et al.
Holanda et al., cohort4
Holanda et al., cohort1
Holanda et al., cohort2
Mesbahi et al., cohort2
Holanda et al., cohort3
Soodabeh et al., cohort2
Soodabeh et al., cohort1
Cappell et al., cohort1
Cappell et al., cohort2
Standard Error
Mean

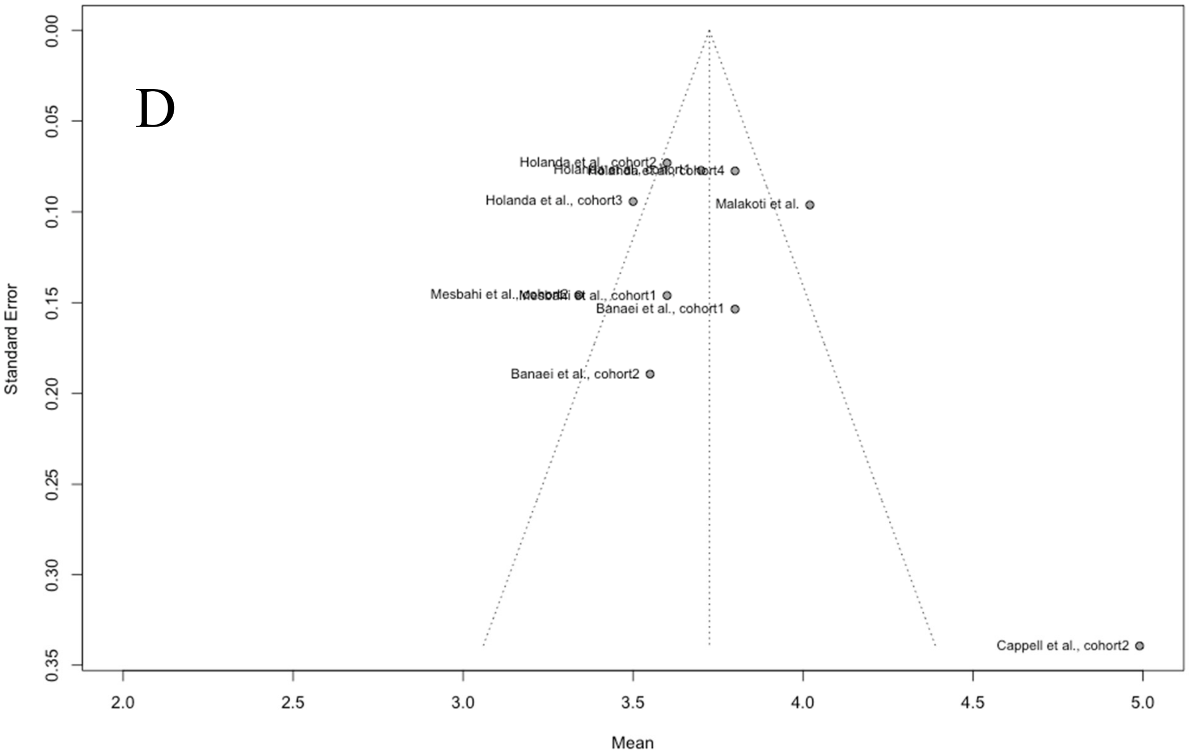
D
Holanda et al., cohort2
Holanda et al., cohort3
Malakoti et al.
Mesbahi et al., cohort1
Banaei et al., cohort1
Banaei et al., cohort2
Cappell et al., cohort2
Standard Error
Mean

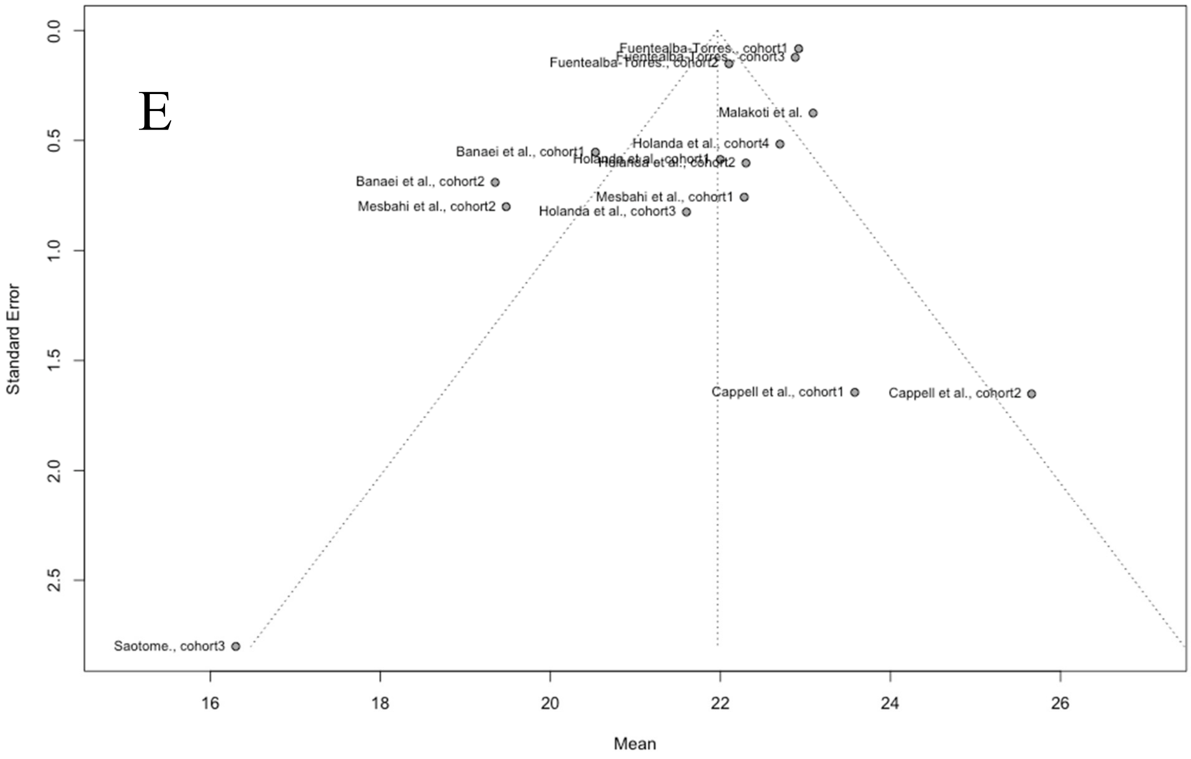
E
Fuentealba-Torres., cohort1
Fuentealba-Torres., cohort3
Fuentealba-Torres., cohort2
Malakoti et al.
Holanda et al., cohort4
Banaei et al., cohort1
Holanda et al., cohort1
Holanda et al., cohort2
Banaei et al., cohort2
Mesbahi et al., cohort1
Mesbahi et al., cohort2
Holanda et al., cohort3
Cappell et al., cohort1
Cappell et al., cohort2
Saotome., cohort3
Standard Error
0.0
0.5
1.0
1.5
2.0
2.5
16
18
20
22
24
26
Mean

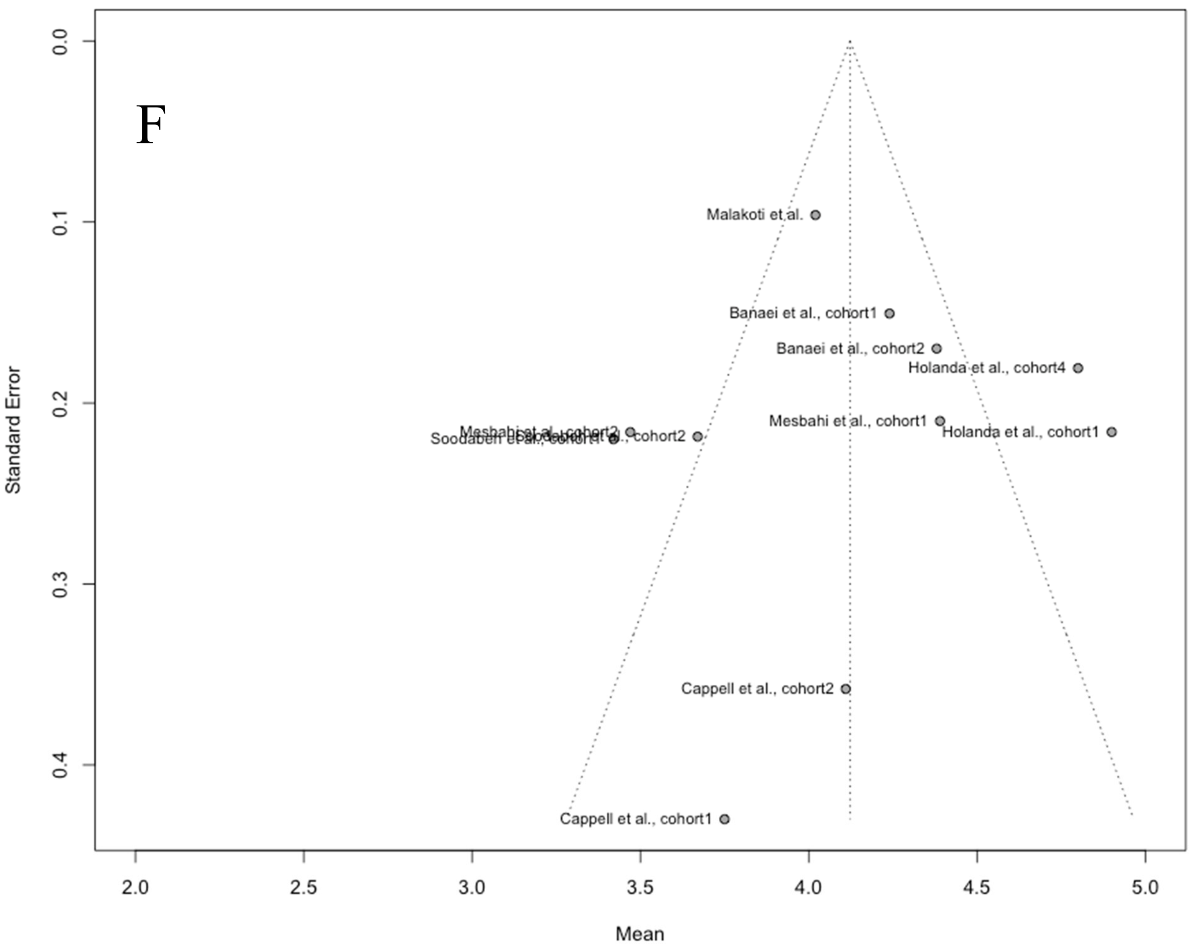
F
Malakoti et al.
Banaei et al., cohort1
Banaei et al., cohort2
Holanda et al., cohort4
Mesbahi et al., cohort1
Holanda et al., cohort1
Cappell et al., cohort2
Cappell et al., cohort1
Standard Error
0.0
0.1
0.2
0.3
0.4
2.0
2.5
3.0
3.5
4.0
4.5
5.0
Mean

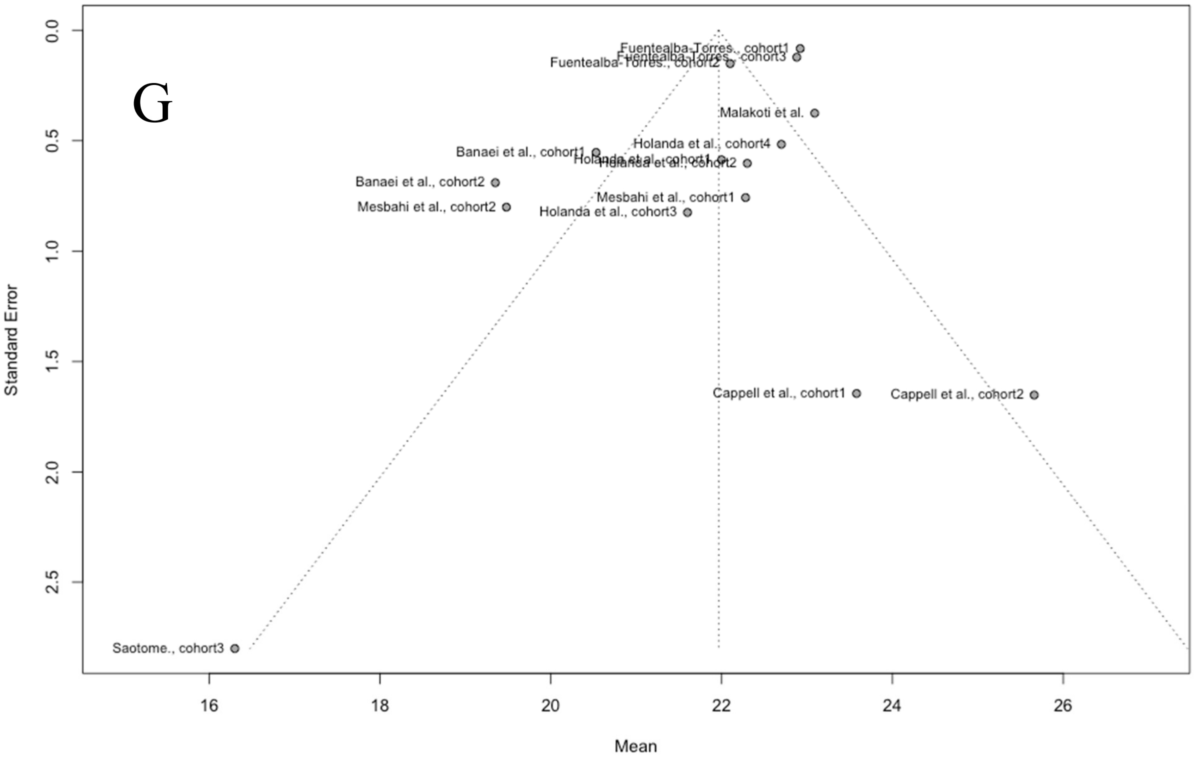

Supplementary Figure S8. Funnel plots exploring publications bias in studies reporting arousal (A), desire (B), orgasm (C), lubrication (D), pain (E), satisfaction (F), and overall sexual function (G).

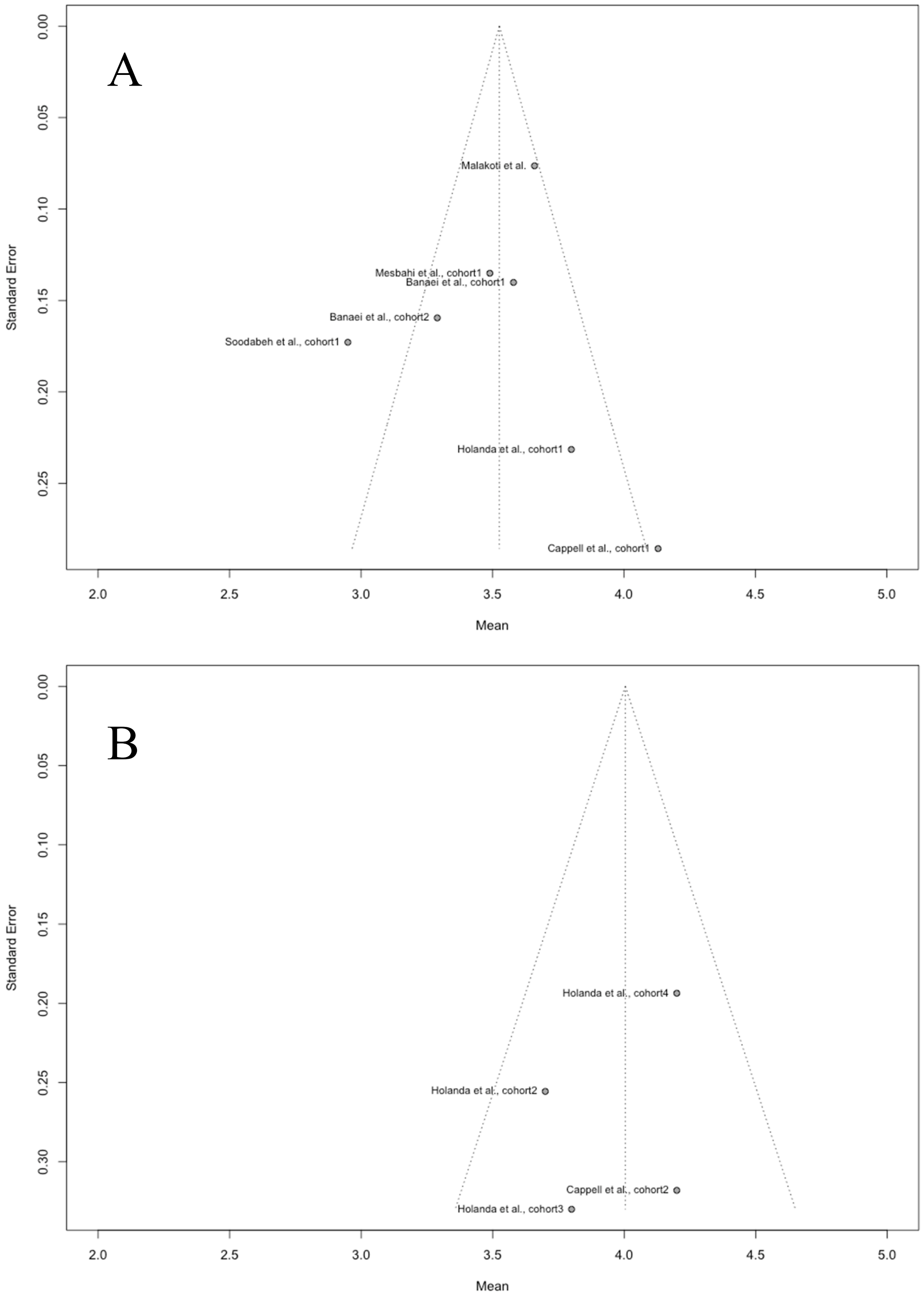

Supplementary Figure S9. Funnel plots depicting publication bias for studies on arousal in women choosing (A) exclusive and (B) complimented breastfeeding

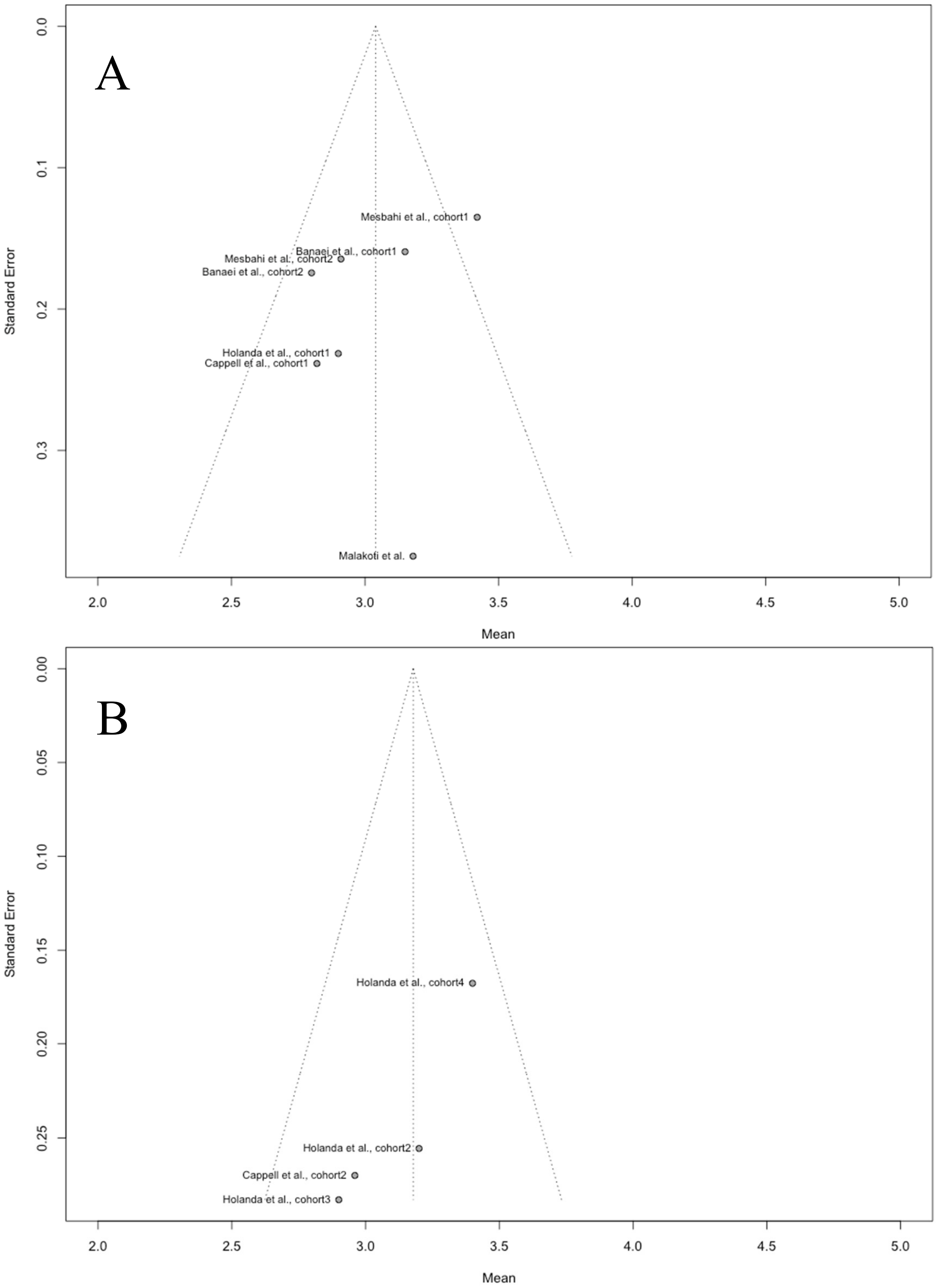

Supplementary Figure S10. Funnel plots depicting publication bias for studies on desire in women choosing (A) exclusive and (B) complimented breastfeeding

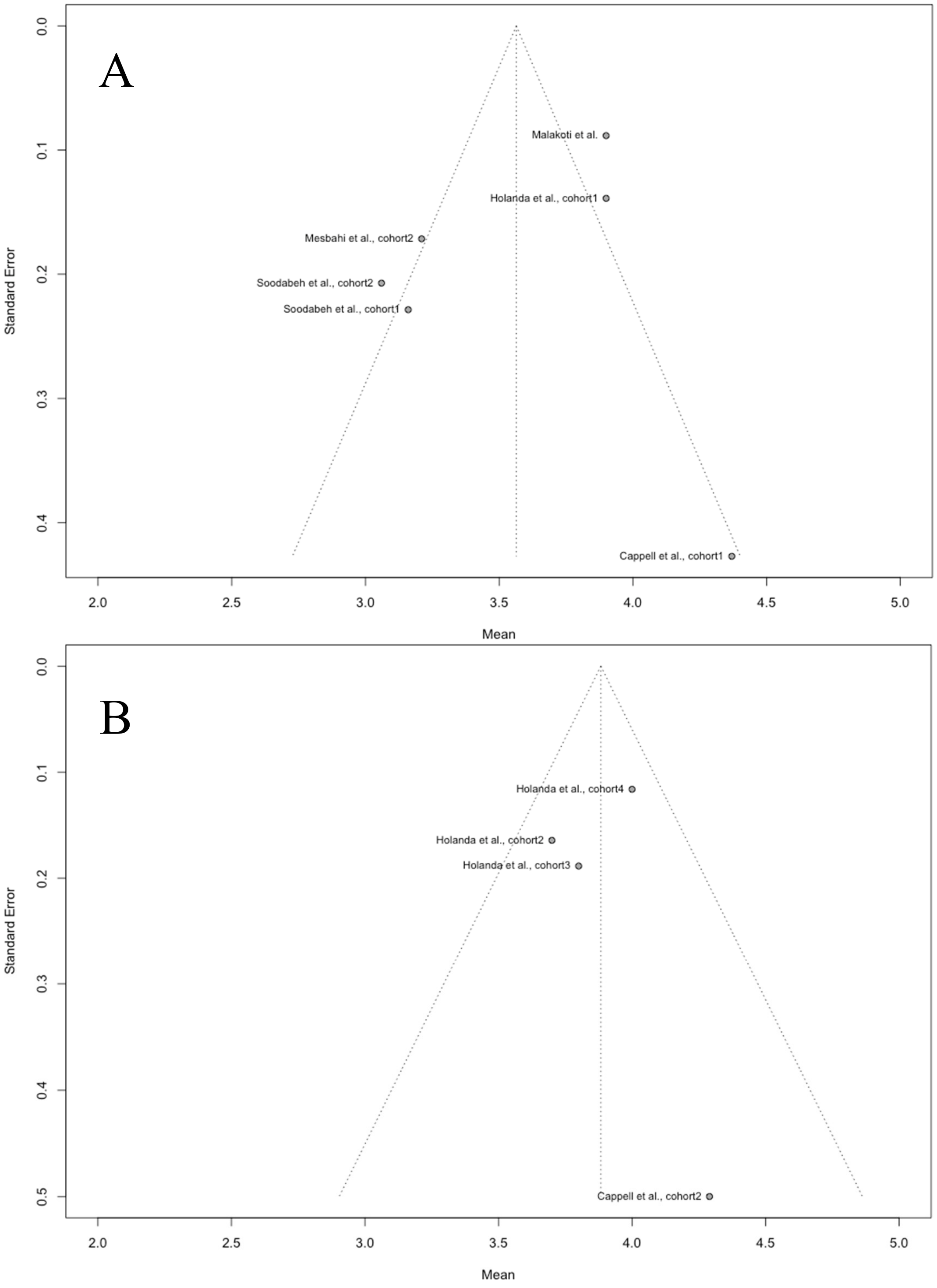

Supplementary Figure S11. Funnel plots depicting publication bias for studies on orgasm in women choosing (A) exclusive and (B) complimented breastfeeding

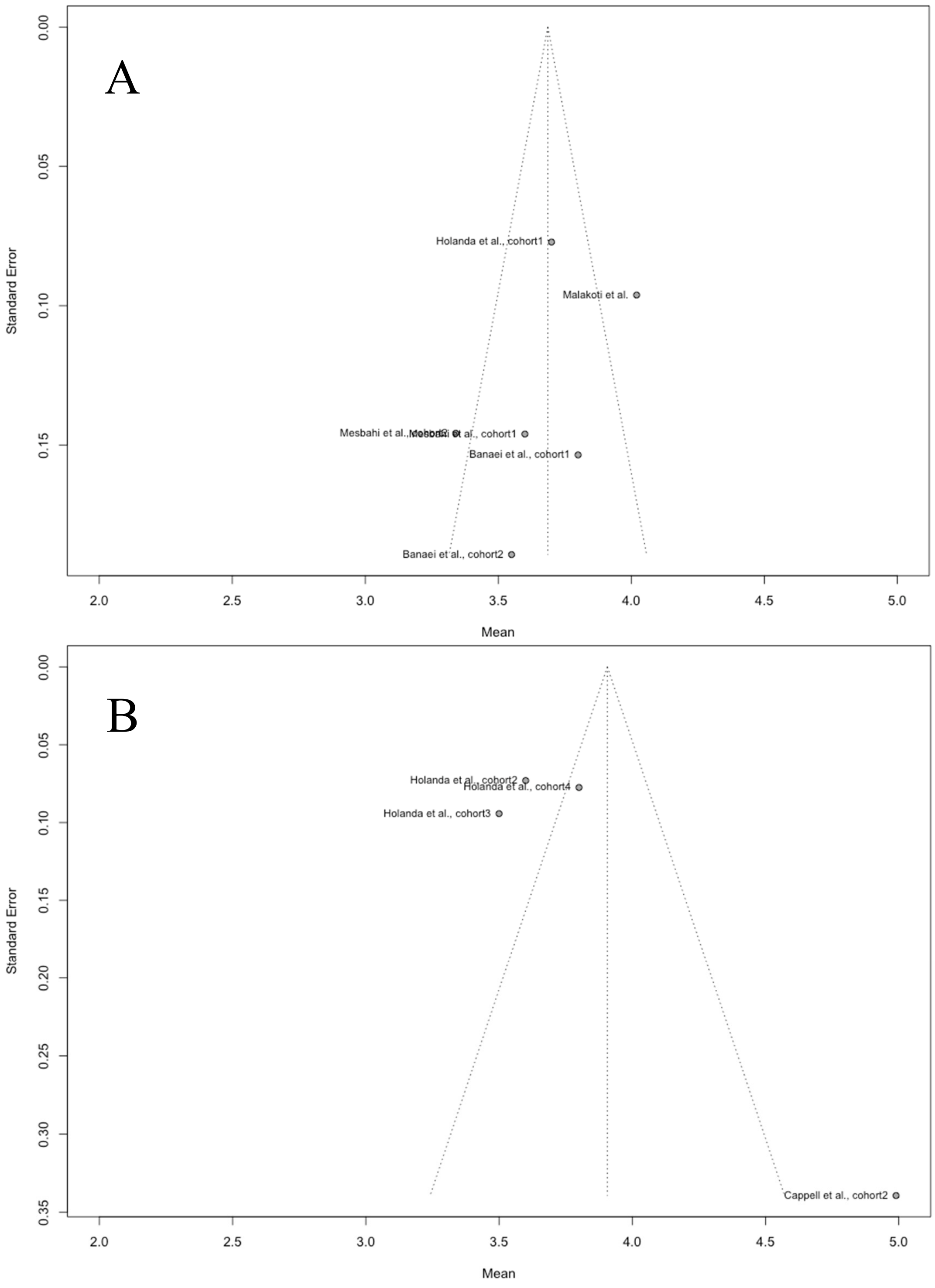

Supplementary Figure S12. Funnel plots depicting publication bias for studies on lubrication in women choosing (A) exclusive and (B) complimented breastfeeding

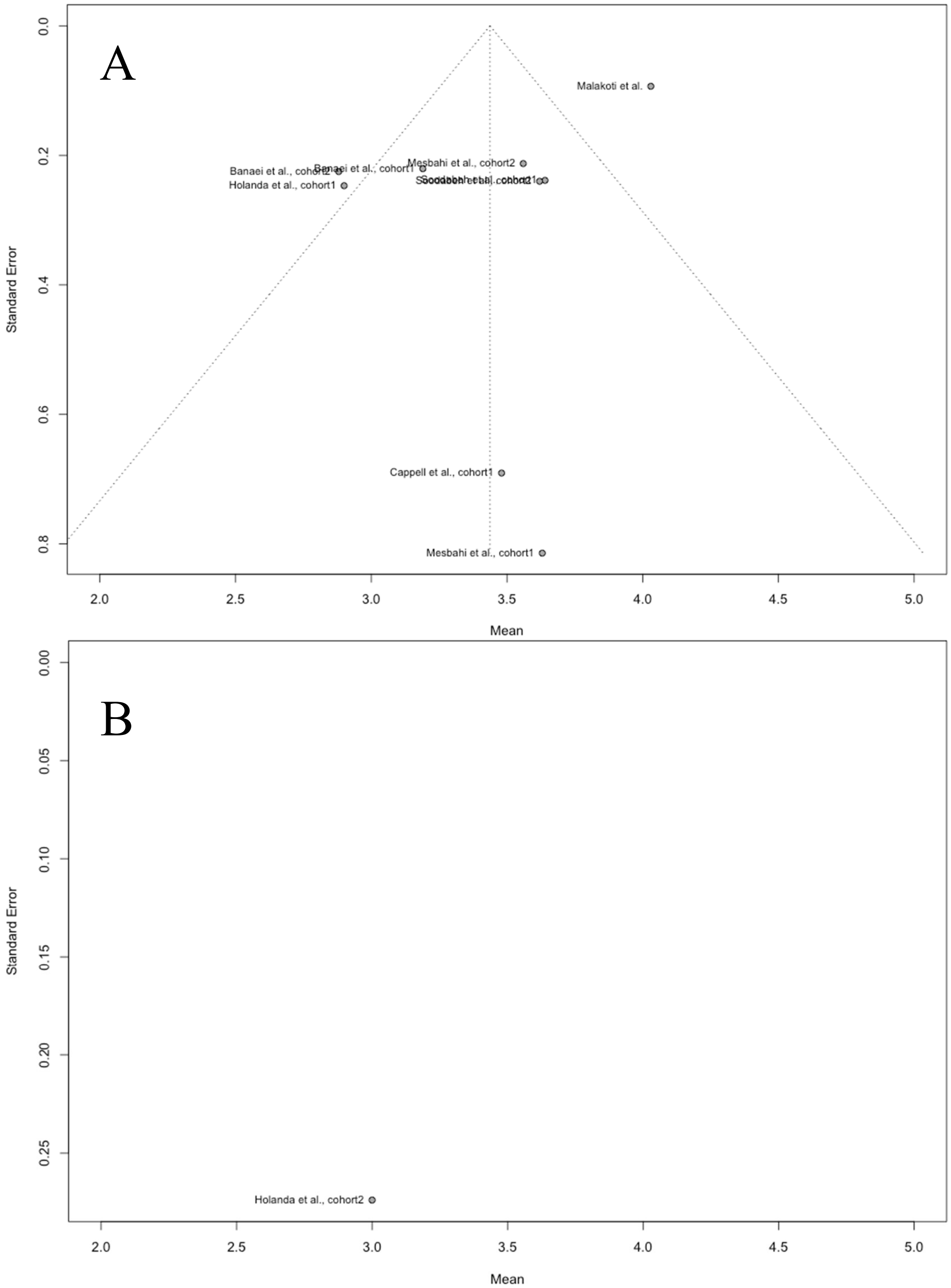

Supplementary Figure S13. Funnel plots depicting publication bias for studies on pain in women choosing (A) exclusive and (B) complimented breastfeeding

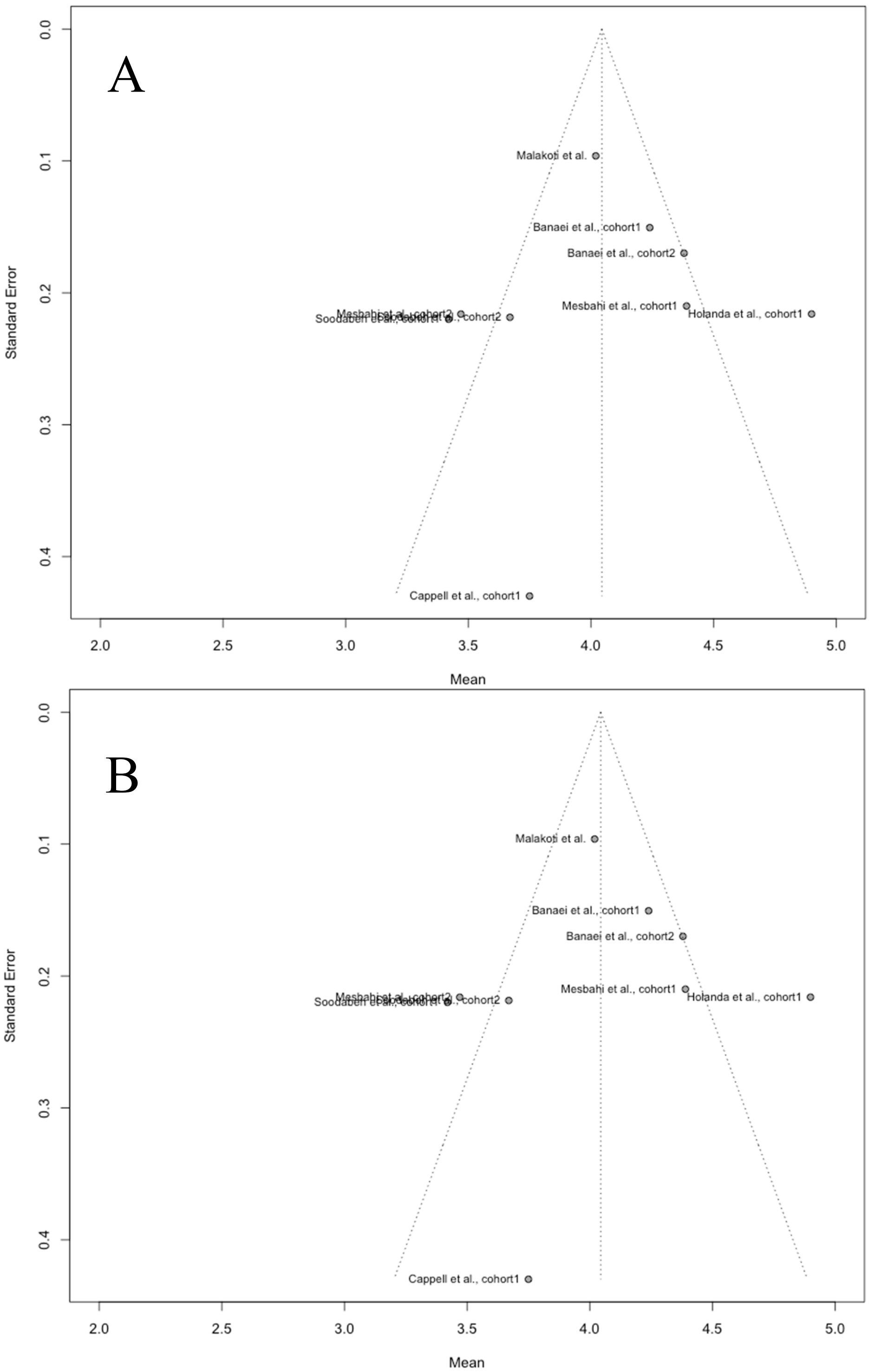

Supplementary Figure S14. Funnel plots depicting publication bias for studies on satisfaction in women choosing (A) exclusive and (B) complimented breastfeeding

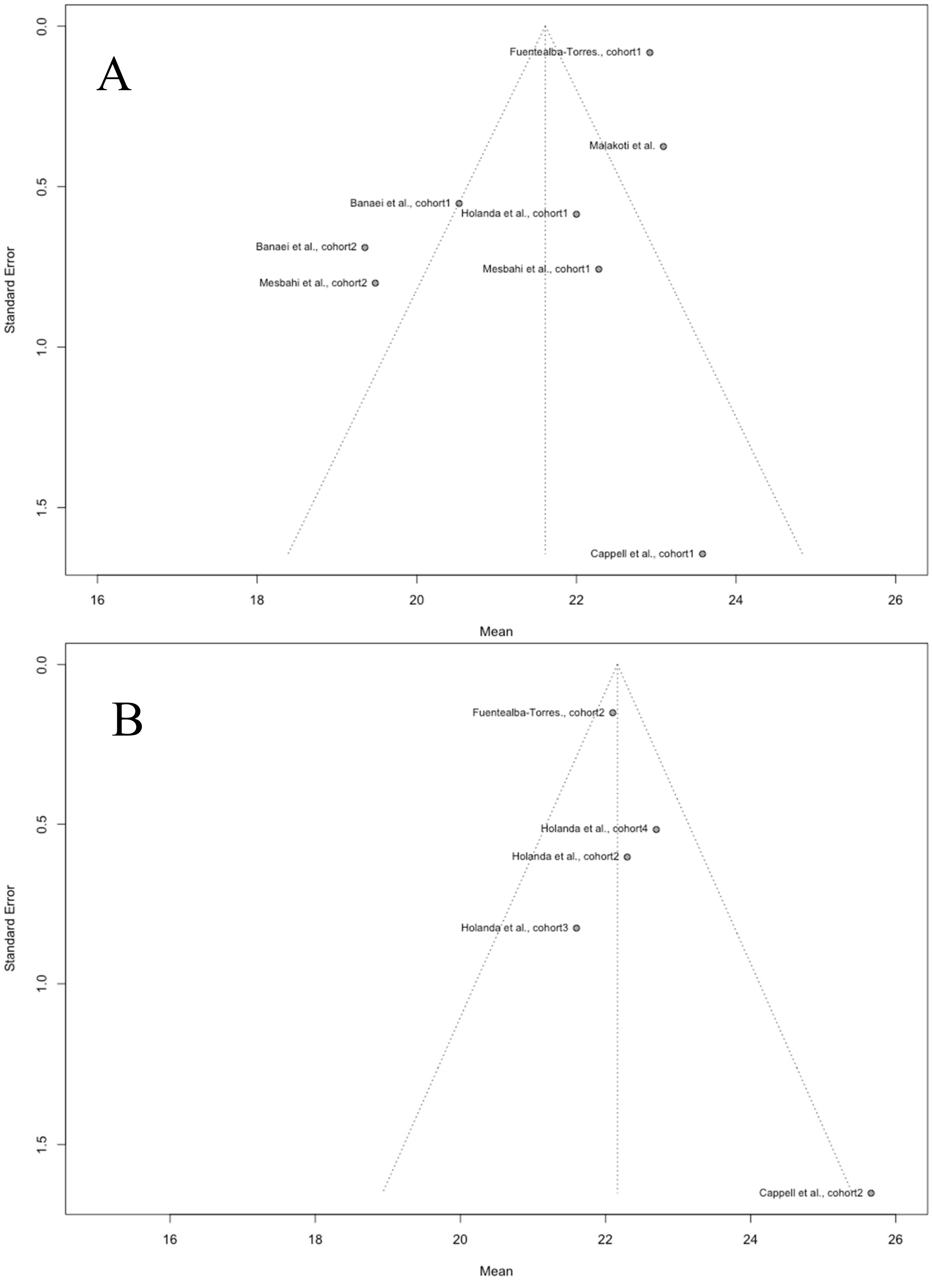
A
Fuentealba-Torres., cohort1
Malakoti et al.
Banaei et al., cohort1
Holanda et al., cohort1
Banaei et al., cohort2
Mesbahi et al., cohort1
Mesbahi et al., cohort2
Cappell et al., cohort1
Standard Error
Mean
B
Fuentealba-Torres., cohort2
Holanda et al., cohort4
Holanda et al., cohort2
Holanda et al., cohort3
Cappell et al., cohort2
Standard Error
Mean

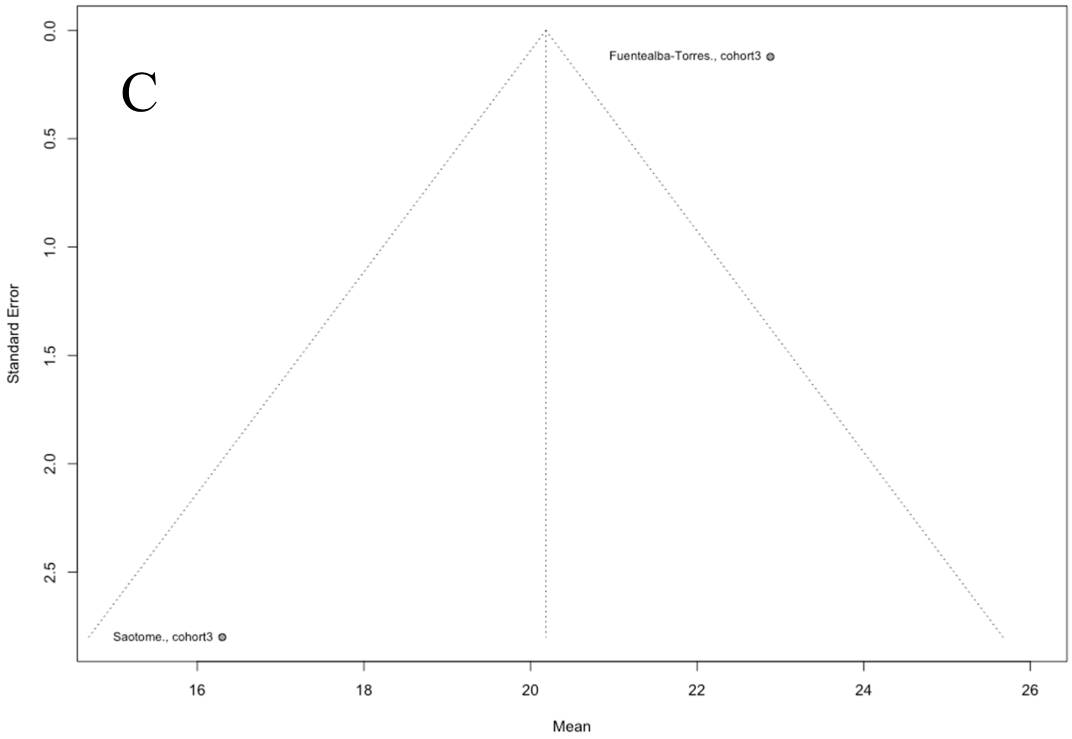

Supplementary Figure S15. Funnel plots depicting publication bias for studies on overall FSFI score in women choosing (A) exclusive, (B)complimented breastfeeding, and (C) bottle feeding
